# Integration of small RNA, degradome, and transcriptome sequencing data illustrates the mechanism of low phosphorus adaptation in *Camellia oleifera*

**DOI:** 10.3389/fpls.2022.932926

**Published:** 2022-08-01

**Authors:** Juanjuan Chen, Xiaojiao Han, Sicheng Ye, Linxiu Liu, Bingbing Yang, Yongqing Cao, Renying Zhuo, Xiaohua Yao

**Affiliations:** ^1^State Key Laboratory of Tree Genetics and Breeding, Chinese Academy of Forestry, Hangzhou, China; ^2^Key Laboratory of Tree Breeding of Zhejiang Province, Research Institute of Subtropical Forestry, Chinese Academy of Forestry, Hangzhou, China; ^3^Forestry Faculty, Nanjing Forestry University, Nanjing, China

**Keywords:** *Camellia oleifera*, low phosphorus stress, integration analysis, miRNA, co-expression network

## Abstract

Phosphorus (P) is an indispensable macronutrient for plant growth and development, and it is involved in various cellular biological activities in plants. *Camellia oleifera* is a unique high-quality woody oil plant that grows in the hills and mountains of southern China. However, the available P content is deficient in southern woodland soil. Until now, few studies focused on the regulatory functions of microRNAs (miRNAs) and their target genes under low inorganic phosphate (Pi) stress. In this study, we integrated small RNA, degradome, and transcriptome sequencing data to investigate the mechanism of low Pi adaptation in *C. oleifera*. We identified 40,689 unigenes and 386 miRNAs by the deep sequencing technology and divided the miRNAs into four different groups. We found 32 miRNAs which were differentially expressed under low Pi treatment. A total of 414 target genes of 108 miRNAs were verified by degradome sequencing. Gene ontology (GO) functional analysis of target genes found that they were related to the signal response to the stimulus and transporter activity, indicating that they may respond to low Pi stress. The integrated analysis revealed that 31 miRNA–target pairs had negatively correlated expression patterns. A co-expression regulatory network was established based on the profiles of differentially expressed genes. In total, three hub genes (*ARF22*, *WRKY53*, and *SCL6*), which were the targets of differentially expressed miRNAs, were discovered. Our results showed that integrated analyses of the small RNA, degradome, and transcriptome sequencing data provided a valuable basis for investigating low Pi in *C. oleifera* and offer new perspectives on the mechanism of low Pi tolerance in woody oil plants.

## Introduction

Phosphorus (P) is an indispensable macronutrient required for plant growth and development and is involved in energy transfer, metabolic regulation, protein activation, and other cellular biological activities in plants (Wang Y. et al., 2021; Wang Z. et al., 2021; Navarro and Munné-Bosch, 2022). Inorganic phosphate (Pi) in soil exists mainly in the form of organophosphate compounds and inorganic Pi (HPO_4_^2–^ and H_2_PO_4_^–^) (Lidbury et al., 2022). Although some soils contain high levels of Pi, the low solubility and high adherence of Pi in soil lead to a lack of available Pi, which is one of the factors that limits crop yield (Crombez et al., 2019). To maintain high crop production, an excessive amount of phosphate fertilizer is often applied to the soil, leading to severe water eutrophication (Conley et al., 2009; Johnston et al., 2014). Moreover, the already insufficient phosphate rock resources are declining and speculated to deplete within 50–100 years (Cordell et al., 2009).

Plants maintain cellular Pi homeostasis by coordinating Pi acquisition, storage, translocation, and remobilization (Fan X. et al., 2021; Jia et al., 2021; Wang Z. et al., 2021). In response to low Pi environments, plants produce various Pi starvation responses to survive. The Pi starvation response is modulated by gene regulatory networks that involve hundreds of Pi transporters and Pi starvation-induced genes (Rubio et al., 2001; He et al., 2015; Wang F. et al., 2018). Pi starvation-induced genes include transcription factors, signal molecules, and other upstream regulators. In *Arabidopsis*, nine phosphate transport 1 (*Pht1*) genes were involved in the absorption of Pi from the soil and the redistribution of Pi in plants (Smith et al., 2003). Phosphate starvation response 1 (*AtPHR1*) and other related transcription factors play vital roles in regulating the Pi starvation response. In the presence of Pi, SYG1/PHO81/XPR1 (*SPX1*) shows high binding affinity to *PHR1*, which inhibits the interaction of *PHR1* with its conserved P1BS motif. However, in the absence of Pi, the binding affinity of *SPX1* to *PHR1* is reduced, allowing the PHR1–P1BS interaction and subsequent induction of transcription to take place (Liu N. et al., 2018; Li et al., 2021). For example, in rice (*Oryza sativa*), *OsSPX1* and *OsSPX2* interact with *OsPHR2* and repress the activity of *OsPHR2* in a similar way to that in *Arabidopsis* (Wang et al., 2014; Tyagi et al., 2021). The phosphorus-responsive transcription factors *OsPHR1/2/3* are located in the core of the mycorrhizal symbiotic transcription regulatory network in plants under Pi starvation (Shi et al., 2021).

Recently, Pi starvation-induced genes, mainly those that encode signaling molecules and transcription factors, have been studied extensively in *Arabidopsis* and rice (Sun et al., 2012; Wang Q. et al., 2018). MicroRNAs (miRNAs) have also been shown to play crucial roles in regulating the Pi starvation response at the posttranscription level (Li et al., 2010; Wang S. et al., 2021). A large number of differentially expressed miRNAs involved in the response to low Pi stress have been identified by using small RNA (sRNA) sequencing. In soybean (*Glycine max*), 36 differentially expressed miRNAs involved in the response to Pi starvation were identified (Xu et al., 2013). In poplar (*Populus tomentosa*), three novel and 65 known miRNAs were found to respond dynamically to Pi fluctuations, and *miR167*, *miR171*, *miR394*, and *miR857* participated in low Pi stress response (Bao et al., 2019). The functions of miRNAs in the response to Pi starvation have also been investigated. In *Arabidopsis*, *miRNA399* was the first shown to regulate the Pi stress response. The expression of *miRNA399* was upregulated when Pi was deficient and decreased quickly when Pi was sufficient (Fujii et al., 2005). Subsequently, six *miRNA399* genes (*miRNA399A–F*) in *Arabidopsis* were found to be induced differently under low Pi stress, and three genes (phosphate transporter 1;7, DEAD box, and ubiquitin E2 conjugase) were predicted to be regulated by *miRNA399*, but only ubiquitin E2 conjugase (*UBC24*) was confirmed to be a target (Allen et al., 2005). In *miRNA399*-overexpressed *Arabidopsis* lines, the expression level of *UBC24* mRNA was reduced significantly (Pegler et al., 2021). Several conserved miRNA families and some novel miRNAs have also been shown to respond to Pi starvation in various plant species. In wheat (*Triticum aestivum*), *TaemiR408* was shown to be of great importance for adaptation to Pi starvation, and its overexpression in tobacco (*Nicotiana tabacum*) increased Pi accumulation upon Pi deprivation by improving Pi absorption (Bai et al., 2018). In Pi-deprived plants, *TamiR1139* overexpression in wheat enhanced the phenotype, biomass, photosynthesis, and Pi acquisition, which suggested that *TamiR1139* was vital in plant Pi starvation tolerance by transcriptionally regulating the target genes and modulating the Pi stress–defense processes (Liu Z. et al., 2018). In *alfalfa* (*Medicago sativa*), under Pi deficiency, the expression of *miR399* and *miR398* was upregulated and the expression of *miR159*, *miR156*, *miR171*, *miR160*, and *miR2111* was downregulated (Li et al., 2018). In the woody tree *Betula luminifera*, the functions of *miR395*, *miR397*, *miR169*, and *miR399* in response to Pi starvation were hypothesized to affect downstream biological processes (Zhang et al., 2021). In brief, the functions of Pi-responsive miRNAs have been studied mainly in herbaceous plants. Many of these miRNAs are species-specific and regulate biological processes by binding to their target genes.

*Camellia oleifera* is a unique high-quality woody oil plant that grows in the hills and mountains of southern China and has a long history of cultivation and consumption. Tea oil is a high-quality edible oil rich in nutrients. The unsaturated fatty acid content of the oil is approximately 80%, and it is easily absorbed and digested by the human body, thus giving it the name “Oriental Olive Oil [*sic*]” (Shi et al., 2020). Suitable distribution areas of *C. oleifera* have red, yellow, or yellow-brown soil with pH values of 4.5–6.5; however, mineral nutrient deficiencies are common in these areas (Li et al., 2019). Pi deficiency reduces *C. oleifera* biomass and Pi accumulation and also affects germination and fruiting (Wu et al., 2019). Until now, research on the adaptation of *C. oleifera* to low Pi has focused on the physiological aspects, and few reports focused on miRNAs (Zhou et al., 2020; Sun et al., 2021). The molecular mechanism of low Pi adaptation in *C. oleifera* requires further investigation to explain its resistance to low Pi. In this study, we aimed to identify miRNAs and their target genes that respond to under low Pi stress in the *C. oleifera*. For this, we combined the sRNA, degradome, and transcriptome sequencing data and generated an integrated resource for recognizing pivotal regulatory miRNA–target gene interactions in plants under low Pi stress. Our results will provide genetic resources for cultivating improved *C. oleifera* varieties that can adapt to low Pi environments and also provide a theoretical foundation for further alleviating the problems of Pi deficiency and environmental pollution.

## Materials and methods

### Plant materials and low phosphate stress treatment

*Camellia oleifera* “changlin166” cuttings (semi-lignified shoots and approximately 20 cm long) were collected from the Dongfanghong Forestry Farm in Jinhua City, Zhejiang Province, China, in June 2019. The cuttings from one single genotype were asexually propagated and cultivated and then grown in an artificial climate culture room under controlled conditions (25–28°C, 16-h light, 8-h dark). The experiment was performed in March 2021. First, individual plastic pots (19 × 11 cm) were filled with sand and placed in a climate chamber. Before beginning the treatment, the seedlings were irrigated every 3 days with a 1/2-strength Hoagland nutrient solution (Coolaber, China). Then, similarly developed plants were treated as follows: (1) control, 1/2-strength phosphorus-free Hoagland nutrient solution supplemented with 1 mM KH_2_PO_4_ and (2) low Pi treatment, 1/2-strength phosphorus-free Hoagland nutrient solution plus 5 μM KH_2_PO_4_. The pH of the nutrient solution was adjusted to 5.8 ± 0.1 by adding diluted HCl or NaOH. Then, 30 uniformly developed plants were divided into five groups, and a completely randomized block design with three replicates was used. Their roots were sampled at CK (control), 1, 3, 7, and 30 days, respectively. Fine powder (100 mg dry weight) was digested in a mixture of 5 mL 98% H_2_SO_4_ and 1 mL H_2_O_2_, and the P content was determined spectrophotometrically at 700 nm based on the molybdenum blue method (Sun et al., 2012). ATPase activity in the roots was analyzed using the modified p-nitrophenyl phosphate (NPP) method (Liu et al., 2004). Samples from the same batch were used for small RNA, transcriptome, and degradome high-throughput sequencing.

All collected root samples were frozen quickly in liquid nitrogen and stored at –80°C. Total RNA was extracted using the Total RNA Purification Kit (NORGEN, Thorold, ON, Canada). The RNA was treated with DNase I (Takara, Dalian, China) to remove any genomic DNA contamination. The integrity and concentration of RNA were measured by denaturing 1.2% (p/v) agarose gel electrophoresis and using a 2100 Bioanalyzer (Agilent Technologies, Santa Clara, CA, United States), respectively. The RNA concentrations of *C. oleifera* were 305.24–610.30 ng⋅μL^–1^. Each sample had an RNA integrity number > 8.0.

To construct cDNA libraries for transcriptome sequencing, the mRNA was obtained from the total RNA using Oligo (dT) magnetic beads and fragmented. First-strand cDNA was synthesized using six random hexamer primers and reverse transcriptase. Second-strand cDNA was synthesized using second-strand buffer, DNA polymerase I, RNase H, and RNase-free deionized water. This was followed by end-repairing of the double-stranded cDNA, and an “A” residue was added to the 3′ end. Then, specific sequencing adapters were attached to both ends of the DNA fragments. The ligated cDNA fragments were purified by gel-tipping (generally, 200 500-bp-long fragments are recovered), and high-fidelity enzymes were used to amplify the sequencing libraries. For sequencing libraries, quality control of raw data, adapters, and trimers and cleanup of low-quality reads were performed by FastQC software. The final products were loaded onto an Illumina HiSeq™ 2500 platform for transcriptome sequencing.

For sRNA sequencing, RNA was isolated from three replicates at five time periods and used to construct 15 sRNA libraries. Gel separation technology was used to collect 18- to 30-nt or 18- to 40-nt fragments in the samples, and adapters were ligated to their 5′ and 3′ ends. The fragments were used for cDNA synthesis, followed by PCR amplification and single-strand separation. The obtained single-stranded cDNA will be circularized and digested. The final products were sequenced on an Illumina HiSeq™ 2500 platform.

Library construction was performed as described previously (German et al., 2008). Raw reads (single-end; 50 bp) with adapter or primer contamination were removed using Trimmomatic V0.35 (Bolger et al., 2014) to obtain clean degradome reads, which were then mapped to the *C. oleifera* transcriptome sequencing data.

### Analysis of transcriptome sequencing data

To ensure that the reads were of high enough quality, the low-quality raw sequencing data were removed and clean reads were obtained. Trinity^1^ with default parameters was used to evaluate the unigene sets obtained by assembling clean reads. Then, the unigenes were searched against the NCBI non-redundant (nr^2^), Kyoto Encyclopedia of Genes and Genomes (KEGG^3^), and Gene Ontology (GO^4^) databases to obtain protein functional annotations and metabolic pathway information (Ünlü et al., 2021). Using the assembled unigenes as the reference, the reads in each sample were mapped to the reference sequences to obtain the read coverage of each unigene in each sample, and then the fragments per kilobase of exon model per million mapped fragments (FPKM) normalization formula was applied to normalize the number of enriched fragments. Differentially expressed genes (DEGs) were identified between the different samples using DE-Seq software (Kvam et al., 2012). A gene was considered to be differentially expressed with |log2(foldchange)| > 1 and *p*-value ≤ 0.05.

### Analysis of the sRNA sequencing data

Adapters, low-quality sequences, regions of low complexity, and common RNA families (tRNA, rRNA, snRNA, and snoRNA) were removed using RFam.^5^ Clean and unique reads were aligned to the miRBase database (v22.0)^6^ using BLAST to identify known miRNAs (Ling et al., 2014). The parameters for known and novel miRNAs and their precursor structure identification were referenced by Axtell and Meyers (2018). The sRNA sequencing data from 15 samples have been deposited in the NCBI SRA database^7^ under accession numbers SRR18672953–SRR18672967. Differentially expressed miRNAs with | log2(foldchange)| > 1 and adjusted *p*-values < 0.05 were selected for further analysis.

### Analysis of degradome sequencing data

Adapter and low-quality sequences obtained by sequencing using the Illumina HiSeq™ 2500 platform were removed, and the adaptor and the clean reads were compared with the assembled unigene reference sequences to obtain the degraded mRNA fragments. Then, miRNA-mRNA degradation sites were analyzed using CleaveLand 4.0^8^ (Addo-Quaye et al., 2009). The target genes were divided into five categories (0, 1, 2, 3, and 4) based on their transcript abundance levels. The predicted target genes were searched against the NCBI nr and nt databases and Swiss-Prot to annotate them. The candidate target genes were functionally annotated by GO and KEGG enrichment analyses.

### cDNA and miRNA syntheses and analyses of gene transcription levels

In total, eight miRNA–target genes were randomly selected for fluorescent qPCR analysis. Reverse transcription was performed using an Mir-X™ miRNA First Strand Synthesis Kit (Takara, Dalian, China) and a PrimeScript™ RT Master Mix (Perfect Real Time) Kit (Takara, Dalian, China) according to the manufacturers’ instructions. The expression levels of the miRNAs and their target genes were determined using an Mir-X miRNA qRT-PCR TB Green^®^ Kit (Takara, Dalian, China) and TB Green^®^ Premix Ex Taq™ II (Tli RNaseH Plus) (Takara, Dalian, China), respectively, on a 7300 Real-Time PCR System (Applied Biosystems, CA, United States). Each sample was run in triplicate. The expression levels of the mRNAs and miRNAs were normalized to 18S rRNA and GAPDH, respectively (Zhou et al., 2013), and were calculated using the 2^–ΔΔ*Ct*^ method (Livak and Schmittgen, 2001). The primers for miRNAs and their target genes are listed in Supplementary Table 1.

### Co-expression network construction

Weighted gene co-expression network analysis (WGCNA) is the systematic biology method for analyzing the correlation patterns among differentially expressed genes across different samples (DiLeo et al., 2011; de Jong et al., 2012). A co-expression network analysis package is freely available at: https://horvath.genetics.ucla.edu/html/CoexpressionNetwork/Rpackages/WGCNA (Langfelder and Horvath, 2008). In brief, the expression profiles of all expressed genes under Pi stress conditions were used to construct a co-expression network. A matrix of pairwise correlation coefficients was used to construct a weighted adjacency matrix that contains pairwise connection strengths using the soft threshold method (power = 6) (de Jong et al., 2012). Rank clustering was performed based on the similarity between genes, and then, the genes with consistent expression in the gene cluster tree are partitioned into the same module (Zhang and Horvath, 2005). Each module is represented by a color, and gene expression profiles were summarized for each module using module signature genes. The gene expression profiles of each module were summarized by the module eigengene and defined as the first principal component of the module expression levels. The targets of differentially expressed miRNAs were selected as hub genes. To display the hub genes, we used Cytoscape software (v3.9.1)^9^ to produce a gene regulatory network diagram based on the data obtained from the transcriptome and degradome analyses (Zhang et al., 2020). Genes with the same or similar functions were classified into one group by GO annotation.

## Results

### Phosphate deprivation alters physiological characteristics

**FIGURE 1:**
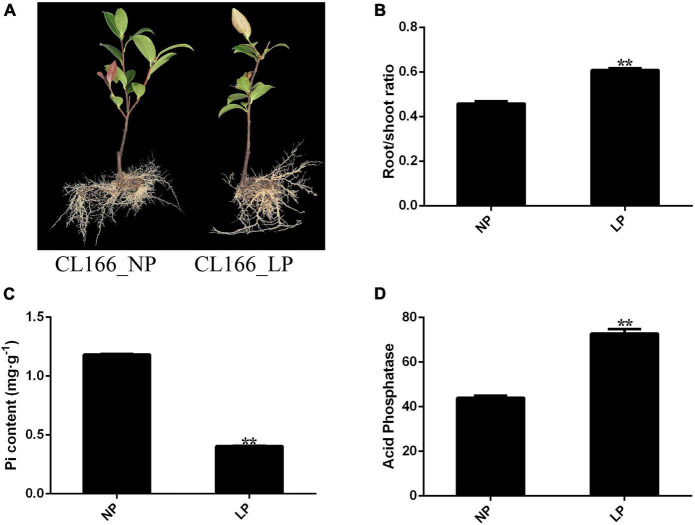
**(A)** Changes in phenotype. **(B)** Root/shoot ratio. **(C)** Pi content. **(D)** Acid phosphate. Bars indicate means ± SE (*n* = 4). *P*-values were obtained from *t*-tests between LP and NP condition. **P < 0.01.

After 30 days of Pi starvation, the root/shoot ratio was increased significantly in roots, and the Pi content in shoots was decreased significantly. However, ATPase activity in roots increased significantly under Pi deprivation. The seedlings of *C. oleifera* showed attenuated growth and increased root biomass after 30 days of Pi starvation (Figure 1).

### Transcriptome sequencing in *Camellia oleifera* roots under low phosphate treatments

Transcriptome libraries were constructed using the extracted total RNA, and 29,389,514 assembled bases were generated. After removing low-quality reads, the remaining high-quality reads were assembled into 40,689 unigenes. Details of the transcriptome sequencing of *C. oleifera* are shown in Table 1. The unigenes were annotated by sequence alignments to a number of different databases. The BLASTX results identified 27,027 unigenes that were homologs of sequences in the nr database. A total of 23,423 and 18,713 genes were annotated with enriched GO terms and KEGG pathways, respectively. In the low Pi treatment groups, 2,787, 1,657, 1,413, and 4,548 genes were upregulated and 2,666, 1,845, 2,189, and 3,929 genes were downregulated at 1, 3, 7, and 30 days, respectively, compared with their expression levels in the corresponding control groups, and 1,586 differentially expressed mRNAs were common to all the time periods (Supplementary Figure 1). The heatmap of the gene expression level (top 3,000 genes with large expression variation) is shown in Supplementary Figure 2. The FPKM of all unigenes is shown in Supplementary Table 2.

**TABLE 1:** Summary of Illumina transcriptome sequencing for *Camellia oleifera.*

Species	*C. oleifera*
Total assembled bases	29,389,514
Number of genes	40,689
N50 length (bp)	1,045
GC (%)	42.57
GO	23,423
KEGG	18,713
Pfam	20,259
swissprot	19,315
EggNOG	26,203
NR	27,027

### Sequencing and identification of known and novel miRNAs

**FIGURE 2:**
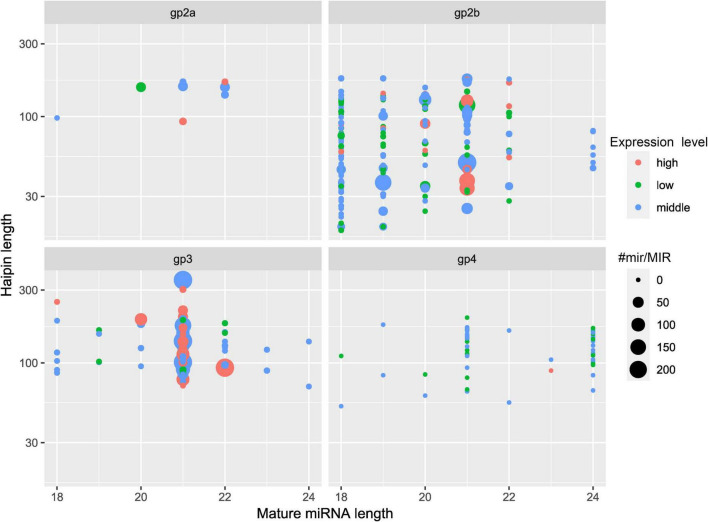
Summary of the classification of the four identified groups of miRNAs in *C. oleifera*. Counts MIRb, the counts of miRNAs from miRBase; expression level, low indicates < 10; middle indicates > 10 but less than average; high indicates over average.

An overview of the obtained raw and clean sequences is given in Supplementary Table 3, and the 18- to 25-nt-long sequences obtained after deleting low-quality sequences are listed in Supplementary Table 4. The number distribution of the sRNAs is shown in Supplementary Figure 3. The length of *C. oleifera* sRNAs was 18–24 nt long, and the 21-nt sRNAs were the most abundant in all 15 libraries. A total of 386 miRNAs were found and divided into four separate groups (Figure 2). A total of 214 pre-miRNAs had the highest degree of similarity with 275 known unique mature miRNAs in 63 miRNA families (Supplementary Figure 4), which had a high degree of similarity compared to other known miRNAs. A total of 70 pre-miRNAs corresponding to 68 mature miRNAs were recognized as novel miRNAs (Supplementary Table 5). Among them, miRNAs of a length of 21 nt made up 38.99% of the known miRNAs, and miRNAs of a length of 24 nt made up 45.59% of the novel miRNAs (Supplementary Table 6). The free energy (dG) and minimum folding free energy index (MFEI) of predicted pre-miRNAs ranged from –17.1 to –93.4 kcal/mol and from 0.9 to 1.7 kcal/mol, respectively (Supplementary Table 7). These characteristics maintain the stability of the hairpin structures of the pre-miRNAs.

### miRNAs that respond to low phosphate in *Camellia oleifera* roots

**FIGURE 3:**
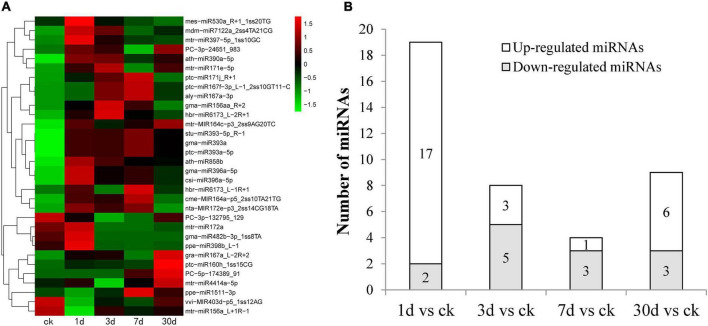
Phosphorus (Pi) deficiency and miRNAs in *C. oleifera*. **(A)** Differential expressed miRNAs in five different low Pi treatment durations (CK, 1, 3, 7, and 30 days) by miRNAs hierarchical clustering. Red indicates higher levels of miRNAs and green indicates lower levels of miRNAs. The names of the samples are shown at the bottom. The original expression values of the miRNAs were normalized using Z-score normalization. The absolute signal intensity ranges from −1.5 to +1.5, with corresponding color changes from green to red. **(B)** The number of differentially expressed miRNAs under Pi deficiency compared with the control.

To identify miRNAs involved in the response to Pi deficiency, the expression levels of miRNAs in the five time periods were analyzed and compared. A total of 32 miRNAs (*p* < 0.05) showed differential expression patterns; among them, 29 were known miRNAs and three were novel miRNAs. A heatmap of differentially expressed miRNAs is shown in Figure 3A. Some members of the same miRNA families had similar expression patterns. For example, two miRNA167s and three miRNA393 were significantly upregulated after low Pi stress for 7 days, and two of the novel miRNAs (*PC-3p-24651_983* and *PC-5p-174389_91*) were upregulated, and one (*PC-3p-132795_129*) was downregulated (Figure 3A). We counted the distribution of 32 differential miRNAs between the control and treatments groups (Figure 3B). The largest number (17) of upregulated miRNAs was found after low Pi treatment for 1 day, and five miRNAs were significantly downregulated after low Pi treatment for 3 days compared with their expression levels in the corresponding controls (Figure 3B). These findings indicate that the duration of the low Pi treatment may have a short-run effect on the miRNA expression levels.

### Prediction of the target genes of the known and novel miRNAs by degradome sequencing

The degradome sequencing generated 61,520,868 raw reads, representing 19,066,404 unique reads from the mixed degradome pool. After removing the low-quality reads and the reads that lacked the adapters, 58,817,780 (95.61% of all reads) sequences were successfully mapped to the 40,689 unigenes. The candidate target genes were divided into five categories, namely, 0, 1, 2, 3, and 4, based on the abundance of degradation sites and transcripts (Addo-Quaye et al., 2009). To select the most credible genes for analysis, we set the alignment score ≤ 4 and category ≤ 2 and obtained 52, 2, and 360 target genes in categories 0, 1, and 2, respectively (Supplementary Table 8). Among the novel miRNAs, 13 miRNAs targeted 21 different genes. In order to predict their authenticity, the target sites of miRNAs and their targets were aligned by psRNATarget^10^ in Supplementary Table 9 (Dai and Zhao, 2011). In addition, t-plots were also constructed to show the cleavage sites for each miRNA–mRNA pair (Supplementary Figure 6).

A total of 414 target genes for 108 miRNAs were identified from the mixed degradome sequencing data; of the 414 target genes, 51 of the miRNAs were predicted to regulate a single gene and 56 of the miRNAs were predicted to regulate two or more genes (Supplementary Table 8). Target genes of 14 novel miRNAs were also obtained by degradome sequencing; eight of them were predicted to regulate a single gene and six novel miRNAs were predicted to regulate two or more genes (Table 2). The length distribution of the novel miRNAs was similar to that of the known miRNAs. We also found that the expression levels of different miRNAs in the same family were different. These results suggest that different miRNAs may have different mechanisms adapted to Pi deficiency in *C. oleifera* roots.

**TABLE 2:** Target genes of 14 novel miRNAs and their functional annotation.

Small RNA	Targets	Alignment score	Cleavage site	Category	Target annotation	Biological process
PC-3p-100894_182	TRINITY_DN30301_c0_g3	4	823	2	LRR receptor-like serine/threonine-protein kinase	Protein kinase activity
PC-3p-132795_129	TRINITY_DN33563_c0_g5	4	297	2	Unknown	Nucleic acid binding
PC-3p-132795_129	TRINITY_DN35456_c0_g2	4	747	2	Calcineurin-binding protein like	–
PC-3p-18140_1384	TRINITY_DN31490_c2_g1	3.5	25	2	Unknown	–
PC-3p-27079_883	TRINITY_DN42730_c0_g2	3	17	2	Unknown	–
PC-3p-30509_767	TRINITY_DN42730_c0_g2	2.5	26	2	Unknown	–
PC-3p-346339_35	TRINITY_DN33230_c0_g2	3	870	2	Branched-chain-amino-acid aminotransferase	–
PC-3p-346339_35	TRINITY_DN42258_c0_g3	4	413	2	Ankyrin repeat-containing protein	Signal transduction
PC-3p-41728_531	TRINITY_DN28460_c0_g1	4	439	2	U3 small nucleolar ribonucleoprotein protein MPP10	Small-subunit processome
PC-3p-41728_531	TRINITY_DN34544_c0_g6	3.5	1733	0	Glycerol-3-phosphate transporter 1 like	Transmembrane transport
PC-3p-41728_531	TRINITY_DN38304_c0_g1	3.5	755	2	Zeaxanthin epoxidase	Secondary metabolite biosynthetic process
PC-3p-44318_495	TRINITY_DN40647_c2_g5	3	71	2	Unknown	–
PC-3p-44318_495	TRINITY_DN42805_c2_g2	4	582	2	Methyl-CpG-binding domain-containing protein	Regulation of transcription
PC-3p-459616_23	TRINITY_DN38590_c0_g4	3	431	2	Hypothetical protein MANES_08G157000	Regulation of transcription
PC-3p-545895_17	TRINITY_DN34459_c0_g1	3.5	144	2	Proteasome subunit alpha type-2-A	Proteasome activity
PC-3p-545895_17	TRINITY_DN45451_c1_g1	3	344	2	Unknown	–
PC-3p-545895_17	TRINITY_DN46149_c1_g4	4	411	2	Unknown	–
PC-3p-66331_303	TRINITY_DN30968_c2_g4	4	101	2	Unknown	–
PC-3p-66331_303	TRINITY_DN44551_c1_g4	4	211	2	AMP deaminese	AMP deaminase activity
PC-3p-90001_209	TRINITY_DN42730_c0_g2	3	15	2	Unknown	–
PC-5p-115327_154	TRINITY_DN24873_c3_g4	2	94	2	Unknown	–
PC-5p-182503_85	TRINITY_DN34544_c0_g6	4	1742	2	Glycerol-3-phosphate transporter 1 like	Transmembrane transport

### Annotation and enrichment analysis of the target genes for miRNAs

**FIGURE 4:**
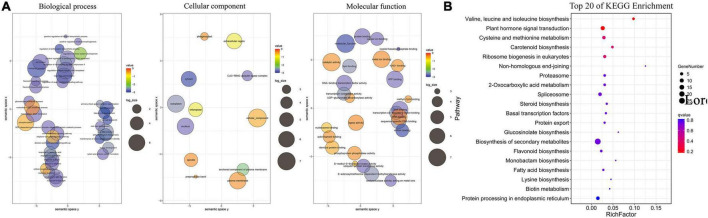
Gene ontology (GO) and Kyoto Encyclopedia of Genes and Genomes (KEGG) functional classification of identified target genes. **(A)** GO enrichment analysis for targets of DE miRNAs under conditions of phosphate (Pi) starvation. **(B)** KEGG Enrichment Analysis for Targets of DE miRNAs under conditions of Pi starvation.

The 414 target genes were annotated with terms under the three main GO categories: biological process, cellular component, and molecular function. The enriched terms were visualized using REVIGO software (Liu et al., 2015) and plotted (Figure 4). Under biological processes, the plant hormone signaling pathways (GO:0009873 ethylene-activated signaling pathway, GO:0009734 auxin-activated signaling pathway, and GO:0009738 abscisic acid-activated signaling pathway) and their response to the stimulus (GO:2000306 positive regulation of photomorphogenesis, GO:0048768 root hair cell tip growth, and GO:0071281 cellular response to iron ion) were enriched, which are consistent with the current understanding of Pi deficiency. Under the cellular component, the nucleus and membrane components were the most enriched term. Under molecular function, DNA binding was the most enriched term, and enzymatic activities (GO:0004842 ubiquitin protein transferase activity, GO:0046522 S-methyl-5-thioribose kinase activity, and GO:0004721 phosphoprotein phosphatase activity) were also enriched, which suggests that these enzymes may play essential roles under low Pi conditions.

The top 20 most enriched KEGG pathways are shown in Figure 4B. Among them, biosynthesis of secondary metabolites (ko01110) and plant hormone signal transduction (ko04075) were the most enriched pathways, which is consistent with GO enrichment results. Plant hormones, such as auxin, salicylic acid, jasmonic acid, ethylene, and gibberellin, are known to be involved in the response to Pi deficiency.

### Correlation analysis of the expression profiles of the miRNAs and their target genes

**FIGURE 5:**
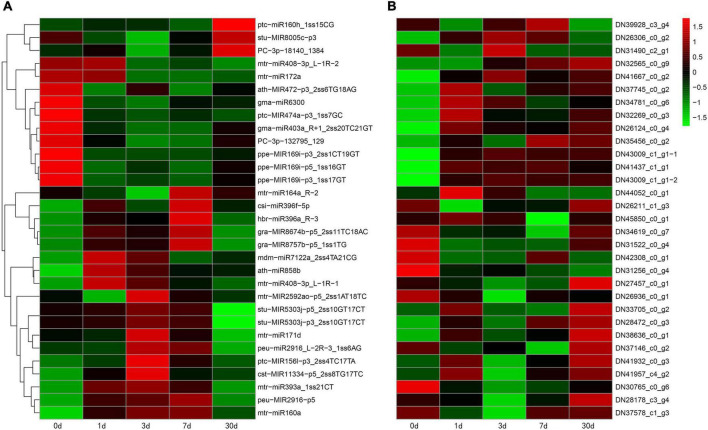
Combined view of expression levels between differentially expressed miRNAs **(A)** and their target genes **(B)** in *C. oleifera* at five different Pi treatment durations. The original expression values of miRNAs and their target genes were normalized by Z-score normalization.

To investigate changes in the whole transcriptome of *C. oleifera* roots under different Pi treatments, the assembled unigenes were used to analyze the differentially expressed genes. In a survey, 48 genes were the targets of 33 miRNAs, which responded to low Pi tolerance (Table 3), and 31 of the miRNA–target genes were negatively correlated with the absolute value of the negative correlation coefficient greater than 0.5 (Figure 5). For example, *ptc-miR160h_1ss15CG* was significantly downregulated and its target auxin response factor 22 (*ARF22*, TRINITY_ DN39928_c3_g4) was significantly upregulated and *mtr*-*miR171d* was upregulated and its target *SCL6* (Scarecrow-like protein, TRINITY_DN38636_c0_g1) was downregulated under Pi deficiency.

**TABLE 3:** Pi deficiency expressed miRNAs and their targets.

miRNA family	miRNA name	Targets	Annotation
miR160	ahy-miR160-5p_L+1_1ss16AT	TRINITY_DN37578_c1_g1	Auxin response factor 10
		TRINITY_DN39928_c3_g4	Auxin response factor like
	ptc-miR160h_1ss15CG	TRINITY_DN37578_c1_g1	Auxin response factor 10
		TRINITY_DN39928_c3_g4	Auxin response factor like
	mtr-miR160a	TRINITY_DN37578_c1_g3	Auxin response factor like
miR4248	aly-MIR4248b-p3_2ss8TC18AC	TRINITY_DN31971_c2_g3	Pumilio 1 like
		TRINITY_DN39965_c1_g1	Mediator of RNA polymerase II transcription subunit 26c
		TRINITY_DN44758_c0_g1	MKI67 FHA domain-interacting nucleolar phosphoprotein
miR399	aqc-miR399	TRINITY_DN34998_c0_g3	Ubiquitin-conjugating enzyme E2 24
	mtr-miR399c	TRINITY_DN25615_c1_g4	–
miR171	mtr-miR171d	TRINITY_DN23484_c2_g1	Scarecrow-like protein 15
		TRINITY_DN27835_c0_g1	Scarecrow-like protein
	ptc-miR171j_R+1	TRINITY_DN35708_c0_g1	Zinc finger SWIM domain-containing protein
	mtr-miR171e-5p	TRINITY_DN43082_c0_g1	PREDICTED: mitochondrial outer membrane protein porin of 34 kDa
	gma-miR171k-3p_2ss11CT19AT	TRINITY_DN38636_c0_g1	Scarecrow-like protein
		TRINITY_DN38636_c0_g5	Scarecrow-like protein 6
miR2592	mtr-MIR2592ao-p5_2ss1AT18TC	TRINITY_DN26826_c0_g2	PREDICTED: uncharacterized protein LOC100245641
		TRINITY_DN26936_c0_g1	WRKY transcription factor
		TRINITY_DN44535_c0_g3	Sucrose phosphate phosphatase
	mtr-MIR2592ay-p5_2ss6AG17CG_2	TRINITY_DN35276_c4_g2	Transcription termination factor like
		TRINITY_DN39805_c0_g1	7-deoxyloganetin glucosyltransferase-like isoform X1
	mtr-MIR2592bj-p5_2ss12TC19AT	TRINITY_DN35421_c0_g1	Eukaryotic translation initiation factor 4B3 like
miR408	mtr-miR408-3p_L-1R-1	TRINITY_DN27457_c0_g1	Ethylene-responsive transcription factor RAP2-3-like isoform X2
		TRINITY_DN31259_c0_g3	Flavanone 3-hydroxylase
	mtr-miR408-3p_L-1R-2	TRINITY_DN26569_c0_g2	Copper-transporting ATPase
		TRINITY_DN32565_c0_g9	Basic blue protein
	mtr-MIR408-p3_1ss13CT	TRINITY_DN32465_c1_g6	Unknown
		TRINITY_DN35836_c1_g2	Transcription factor bHLH83
	stu-miR408b-3p	TRINITY_DN26662_c1_g5	Unknown
miR172	gma-miR172a	TRINITY_DN40809_c0_g1	Ethylene-responsive transcription factor like isoform 2
	gma-miR172b-5p_L-1R+2	TRINITY_DN32888_c2_g5	LMBR1 domain-containing protein 2 A like
	stu-miR172a-3p	TRINITY_DN28507_c2_g5	Ethylene-responsive transcription factor RAP2-7 like
	vvi-MIR172d-p5_2ss7GT17CT	TRINITY_DN25739_c0_g1	Ribosome biogenesis regulatory protein, partial
	mtr-miR172a	TRINITY_DN41667_c0_g2	Ethylene-responsive transcription factor RAP2-7 like
miR169	ppe-MIR169i-p3_1ss17GT	TRINITY_DN38370_c1_g6	General transcription factor 3C polypeptide like
	ppe-MIR169i-p3_2ss1CT19GT	TRINITY_DN41819_c0_g4	Transcription factor HHO3-like
	ppe-MIR169i-p5_1ss16GT	TRINITY_DN26297_c0_g2	ABC transporter C family member 3 like, partial
	ptc-MIR169o-p5_2ss1AC17TC	TRINITY_DN44254_c0_g1	Unknown
miR156	ptc-miR156a_L+1	TRINITY_DN32530_c0_g1	Squamosa promoter-binding-like protein
	ptc-MIR156l-p3_2ss4TC17TA	TRINITY_DN41932_c0_g3	Transcription elongation factor like
	mtr-miR156a_L+1R-1	TRINITY_DN40091_c1_g7	Squamosa promoter-binding-like protein
miR472	ath-MIR472-p3_2ss6TG18AG	TRINITY_DN37745_c0_g2	F-box/FBD/LRR-repeat protein
miR858b	ath-miR858b	TRINITY_DN31256_c0_g4	Myb-related protein
		TRINITY_DN31679_c2_g2	Transcription factor MYB5e
		TRINITY_DN42660_c1_g4	Transcription factor MYB5b
Novel miRNA	PC-3p-346339_35	TRINITY_DN33230_c0_g2	Branched-chain-amino-acid aminotransferase
		TRINITY_DN42258_c0_g3	Ankyrin repeat-containing protein
	PC-3p-41728_531	TRINITY_DN28460_c0_g1	U3 small nucleolar ribonucleoprotein protein MPP10
		TRINITY_DN34544_c0_g6	Glycerol-3-phosphate transporter 1 like
		TRINITY_DN38304_c0_g1	Zeaxanthin epoxidase
	PC-5p-182503_85	TRINITY_DN34544_c0_g6	Glycerol-3-phosphate transporter 1 like

**FIGURE 6:**
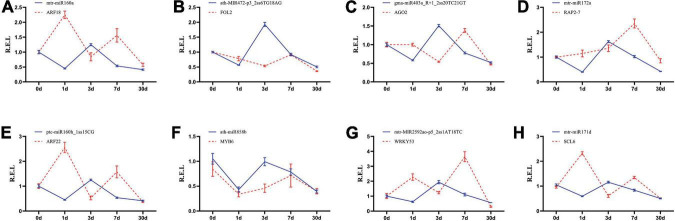
Expression correlation between miRNAs and their targets at five different Pi treatment durations. **(A)** *mtr-miR160a*/*ARF18*; **(B)** *athMIR472p3_2ss6TG18AG*/*FOL2*; **(C)** *gmamiR403a_R* + *1_2ss20TC21GT*/*AGO2*; **(D)** mtr-miR172a/RAP2-7; **(E)** ptc-miR160h_1ss15CG/ARF22; **(F)** *ath-miR858b*/*MYB6*; **(G)** *mtr-MIR2592ao-p5_2ss1AT18TC*/*WRKY53*; **(H)** *mtr-miR171d*/*SCL6*; The blue and red lines indicate miRNAs and the target abundance, respectively, based on the RT-qPCR results.

A RT-qPCR analysis was conducted to verify the expression profile of eight key miRNA–target pairs. The results showed that the expression trends of the eight miRNAs and target genes were similar to those obtained by deep sequencing (Figure 6). The expression levels of the eight miRNA–target pairs were confirmed to be negatively correlated. For example, *mtr-miR160a* and *ptc-miR160h_1ss15CG* were upregulated and then downregulated at the longer time periods, and their target genes showed significant opposite expression patterns. These results suggest that transcription of the target mRNAs may be repressed by the corresponding miRNAs (Figure 6). These results suggest that transcription of the target miRNAs may be repressed by the corresponding miRNAs (Figure 6).

### Gene co-expression network analysis

**FIGURE 7:**
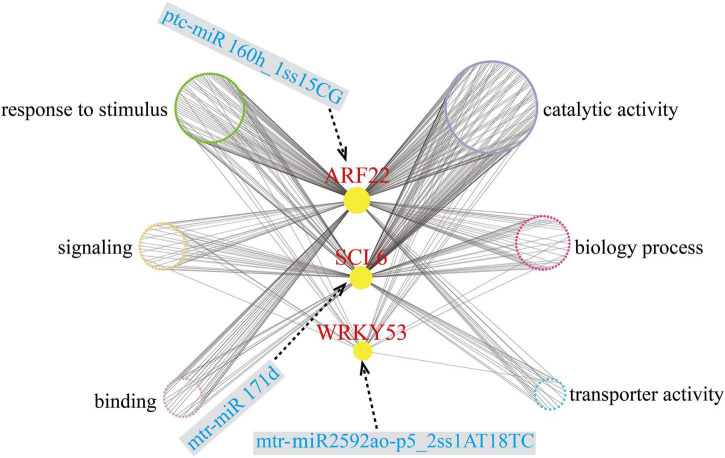
Co-expression subnetwork of *ARF22*, *WRKY53*, and *SCL6*. Red-colored letter denotes key hub gene; blue-colored letter denotes key miRNA; six different colored circles represent six different categories.

After removing unexpressed and lowly expressed genes, we identified 34 gene co-expression modules that contained 40,689 unigenes (Supplementary Figure 5). We constructed a co-expression regulatory network based on different modules (Figure 7). To explore the network connections for the target genes, we focused on three of the differentially expressed target genes (*ARF22*, *ptc-miR160h_1ss15CG* target gene; *WRKY53*, *mtr-miR2592ao-p5_2ss1AT18TC* target gene; and *SCL6*, *mtr-miR171d* target gene), which were hub genes, which may play important roles in low Pi stress. A complete list of the module assignments for the three hub genes is provided in Supplementary Table 10. In the network, the three hub genes *ARF22*, *WRKY53*, and *SCL6* are directly connected with 735, 46, and 272 edge genes, respectively. These hub genes will provide a foundation for future studies.

We also created a subnetwork containing 390 genes that were annotated with six GO terms related to the response to stimulus, signaling, binding, catalytic activity, biological process, and transporter activity (Figure 7); 94, 44, and 20 of these genes were annotated in the response to stimulus, signaling, and transporter activity terms, respectively (Supplementary Table 11). In the subnetwork, there were three signaling-related transcription factors as hub genes that may be involved in Pi uptake, transport, and homeostasis, and four transporter-related members of the ABC family also may promote Pi uptake and transport. When plants experience Pi deficiency, phosphate transporter and vacuolar H^+^ pyrophosphatase together maintain Pi balance.

## Discussion

*Camellia oleifera* is a woody oil plant that grows in southern China. It is frequently exposed to low Pi availability in red soils and has evolved a suite of adaptive mechanisms to cope with conditions of Pi deficiency. Understanding the potential molecular mechanisms is essential for screening germplasm resources resistant to low Pi. The regulation of plant resistance to nutritional deficiency by the miRNA–target gene interactions has been described in several herb plants. However, the molecular mechanisms underlying the responses of woody plants to Pi deficiency are poorly understood. In this study, we performed an integrated analysis to investigate the regulatory network in the roots of *C. oleifera* under Pi deficiency based on miRNA, degradome, and transcriptome sequencing data. Our results showed that the genes targeted by miRNAs play important roles in response to Pi deficiency in the roots. Although our findings provide some understanding of the low Pi adaptation mechanisms, many more studies are needed to gain a comprehensive understanding of the miRNA–target genes of Pi deficiency.

miRNAs have been studied in many plants, but very few studies have been conducted on plants in the family *Theaceae*, which includes *C. oleifera*. In this study, the transcriptome sequences of *C. oleifera* were used as reference sequences for the miRNA and the degradome sequencing analysis. The relationship between miRNAs and target genes was obtained by degradome sequencing. Then, the miRNA–target gene network regulation map showed that the regulation of multiple target genes could be regulated by one miRNA and that one target gene could be regulated by multiple miRNAs. A total of 386 miRNAs were identified and divided into four groups (Figure 2), out of which 284 of them were highly confident miRNAs. Only 14% of them were relatively highly expressed, confirming that high-throughput sequencing can identify miRNAs with low expression levels in plants. The length of mature miRNAs was 18–24 nt (Supplementary Figure 3), which is consistent with previous results in plants such as alfalfa (Li et al., 2018), wheat (Bai et al., 2018), *Betula luminifera* (Zhang et al., 2021), poplar (*Populus tomentosa*) (Bao et al., 2019), and pine (*Pinus massoniana*) (Fan F. et al., 2021).

We identified 32 differentially expressed miRNAs (29 conserved and 3 novel miRNAs) under low Pi stress (Figure 3A). Different members of the same miRNA family had similar expression levels; for example, three *miR393* and two *miR171* family members were significantly upregulated under low-Pi conditions. Similar phenomena were also found in other species. In *Arabidopsis*, *miR393s* play important roles in the root system architecture of Pi deficiency and nitrogen deprivation (Vidal et al., 2010). In *Populus tomentosa*, the expression of *miRNA171e* decreased after 24 h of Pi deficiency and then increased dramatically when Piwas supplied (Bao et al., 2019). In maize (*Zea mays*), under low Pi stress, members of the miR393 family were differentially expressed in the leaves and roots (Gupta et al., 2017). In *Medicago truncatula*, *miR171h* is involved in integrating nutrient homeostasis by regulating the expression of *NSP2* during arbuscular mycorrhizal and root nodule symbiosis (Hofferek et al., 2014). Therefore, *miR393* and *miR171* show different expression patterns in response to low Pi in different plants. In our study, we found two miR167 and two miR396 family members that were significantly upregulated by Pi deficiency. In maize, members of the miR396 family were differentially expressed by Pi starvation, *zma-miR396a* expression was continuously suppressed by Pi starvation, *miR396c* expression was slightly increased during the early phase of low Pi stress, and *zma-miR396d* was significantly upregulated in maize leaves (Nie et al., 2016). We also found two miR156 family members that showed contrary expression patterns in *C. oleifera* roots. *miR156* is a highly conserved miRNA that plays key roles in many biological processes, including developmental and metabolic regulation, immune response, and abiotic stress. In *Arabidopsis*, members of the miR156 family responded to phosphate deprivation and played a potential role in phosphate homeostasis (Hsieh, 2010). Together, these results indicate that miRNAs have diverse functions in plants, and the complex mechanisms need to be further explored in future studies.

We used the transcriptome sequences as reference sequences and analyzed the correlation between miRNAs and their target genes by degradome sequencing. Many target transcripts were identified for the known and novel miRNAs (Han et al., 2016). Mature miRNAs regulate their target mRNAs by forming an miRNA-induced silencing complex that cleaves miRNA or inhibits its translation, thereby negatively regulating the target genes (Sun et al., 2019). miRNAs are known to be involved in abiotic and biotic stresses in plants, and miRNAs can be expressed to act on target genes under abiotic stresses (Cui et al., 2020; Li and Yu, 2021; Ma et al., 2021). We identified 414 genes that were predicted to be targeted by 108 corresponding miRNAs by degradome sequencing (Supplementary Table 8). We also identified 30 differentially expressed target genes that were targeted by 31 miRNAs, and most of these miRNA–target gene pairs had opposite expression patterns (Figure 4), including *ptc-miR160h_1ss15CG*, which was significantly downregulated under Pi deficiency, whereas its target gene *ARF22* (TRINITY_DN39928_c3_g4) was significantly upregulated. However, in *Arabidopsis*, *miR160* and its target genes *ARF16*, *ARF10*, and *ARF17* regulate hypocotyl elongation in a light, brassinazole (BRZ, a BR biosynthesis inhibitor), or paclobutrazol (PAC, a GA biosynthesis inhibitor)-dependent manner (Dai et al., 2021). This finding also indicates that the same miRNA can regulate different target gene pairs and play different roles in different plants.

*miR399* is responsive to Pi starvation and plays important regulatory roles in Pi homeostasis (Pegler et al., 2020, 2021; Peng et al., 2021). In our study, two miR399 members, *miR399* and *miR399c*, which regulated six and 11 mRNAs, respectively, were detected by the analysis of our degradome sequencing data (Supplementary Table 12). However, *miR399* and *miR399c* were not differentially expressed miRNAs, suggesting that *C. oleifera* may have evolved some other miRNAs in response to low Pi stress. In addition, *miR827* was absent in the present sRNA libraries; the possible reason is the lack of its precursor sequence in the draft genome, which led to the loss of *miR827* during evolution or the failed match during sRNA library construction. Similar phenomena were observed in *B. luminifera* and *C. papaya*, in which the *miR827* locus was lost during evolution (Lin et al., 2018; Zhang et al., 2021).

There are 199 miRNA-target genes whose expression levels are negatively correlated (Supplementary Table 13). Overall, three transcription factors as hub genes, *ARF22*, *WRKY53*, and *SCL6*, may play pivotal roles in the regulation of low Pi-responsive genes through a co-expression regulatory network and are critical mediators in regulating various abiotic stresses (Wang X. et al., 2021). In durum wheat (*Triticum turgidum* subsp. *Durum*), *ttu-miR160* was upregulated, and its target *ARF22* was significantly downregulated in roots under chronic and short-term nitrogen stress (Zuluaga et al., 2018). In rice, several miR160s were downregulated in roots and shoots under heat stress (Sailaja et al., 2014). In our study, *ARF22* was targeted by *ptc-miR160h_1ss15CG*, and *ptc-miR160h_1ss15CG* was slightly downregulated at the early stage and increased dramatically after 30 days under low Pi stress, whereas *ARF22* showed opposite expression patterns (Figure 6). *WRKY53* is involved in a complicated transcription factor signaling network that regulates senescence-specific gene expression and may participate in signal transduction in *Arabidopsis* (Miao et al., 2004). Several other WRKY family members were reported to be involved in the low Pi response, such as in an *OsWRKY74*-overexpressing plant that showed significantly enhanced tolerance to Pi starvation, whereas RNAi lines of *OsWRKY74* were sensitive to Pi starvation (Dai et al., 2016). In soybean, *WRKY46* showed an opposite expression pattern to that of *OsWRKY74*. The *GmWRKY46-*overexpressed plants were more sensitive to low Pi stress than the controls, whereas RNAi lines of *GmWRKY46* had significantly enhanced Pi deficiency tolerance (Liu et al., 2022). Previous studies showed that *miR171c*-targeted *SCL6* plays an important role in the control of shoot branch production in *Arabidopsis* (Wang et al., 2010). In soybean, the *miR171*–*SCL6* pair regulated rhizobium symbiosis (Hossain et al., 2019). We found that the *miR171d*–*SCL6* pair may respond to low Pi availability. We consider that these three hub genes and the miRNAs that target them may have major roles in the response to Pi deficiency in *C. oleifera* roots. However, the specific regulatory mechanism needs to be further verified.

## Conclusion

In the study, we performed an integrated analysis of sRNA, degradome, and transcriptome sequencing data and generated a well-rounded resource centered on identifying key regulatory miRNA–target gene pairs that respond to low Pi stress. A total of 40,689 unigenes and 386 miRNAs were identified, and 32 significantly differentially expressed miRNAs were detected. The edge genes of the target hub genes for the low Pi-responsive miRNAs were involved in transporter activity, the response to the stimulus, catalytic activity, signaling, binding, and biological processes. Furthermore, 30 differentially expressed target genes of 31 differentially expressed miRNAs were identified by a concordant analysis of the expression patterns of the miRNA–target gene pairs. A total of 390 co-expressed genes formed a co-expression regulatory subnetwork in which three hub target genes, *ARF22*, *WRKY53*, and *SCL6*, which may play key roles in controlling transcriptomic regulation in response to low Pi stress, were identified. Our results provided a comprehensive understanding of Pi deficiency in *C. oleifera* and will help explain miRNA-mediated molecular mechanisms associated with plant responses to low Pi stress.

## Data availability statement

The original contributions presented in the study are included in the article and Supplementary material. Transcriptome raw reads sequence data are available through the NCBI Sequence Read Archive (BioProject: PRJNA792896; BioSample: SAMN24471059; SRA Accession Number: SRR17365487-SRR17365501, 15 samples). Degradome raw reads sequence data are available through the NCBI Sequence Read Archive (BioProject: PRJNA795525; BioSample: SAMN24731130; SAMN24471059; SRA Accession Number SRR17494566). Small RNA raw reads sequence data are available through the NCBI Sequence Read Archive (BioProject: PRJNA824141; BioSample: SAMN27393800; SRA Accession Number: SRR18672953-SRR18672967, 15 samples).

## Author contributions

JC and XY conceived this project. JC and XH designed experiments and interpreted the results. JC wrote the manuscript. XH provided technical guidance for the experiment. SY, LL, BY, and YC performed the experiments and analyzed the data. RZ and XY provided experimental materials and funds. All authors read and approved the submission of this manuscript.

## Footnotes

1
http://trinityrnaseq.github.io/

2
https://www.ncbi.nlm.nih.gov/refseq/about/non-redundantproteins/

3
https://www.genome.jp/kegg/

4
http://geneontology.org/

5
http://rfam.janelia.org

6
http://www.mirbase.org/

7
https://www.ncbi.nlm.nih.gov/sra/?term=

8
https://github.com/MikeAxtell/CleaveLand4

9
https://cytoscape.org/download.html

10
https://www.zhaolab.org/psRNATarget/analysis?function=3
