# Supplementary material for: Integration of small RNA, degradome, and transcriptome sequencing data illustrates the mechanism of low phosphorus adaptation in Camellia oleifera
Source: Front Plant Sci. 2022 Aug 1;13:932926. doi: 10.3389/fpls.2022.932926 (PMC9377520; doi:10.3389/fpls.2022.932926)
Supplement: Supplementary file 1 [file Data_Sheet_1.ZIP › Supplementary materials/Supplementary Table S1 Gene primers used in qRT-PCR .docx]

1. **mtr-miR160a TGCCTGGCTCCCTGTATGCCA**

**TRINITY_DN37578_c1_g3 Auxin response factor like (ARF18)**

TTTCCTCAGGGCCACGCCGAGCATGCCCTCACAACCGTCGATTTCGGGGCCTTGCCGAGGCTTCCGGCTCTGATCCTCTGCAGAGTTACTGCAGTGAAATTCATGGCCGATGCTGAAACTGATGAAGTTTATGCGAAAATTAGGTTAATTCCTATGAGAAGCAACGAGCTTGATTATGACGATGATGGGGTTTTGGGAAGTAATGGATCCGAATCCCCTGAAAAACCTGCTTCTTTCGCCAAAACATTGACTCAATCGGATGCTAACAACGGTGGTGGATTCTCAGTTCCTCGGTACTGTGCGGAGACTATCTTCCCCCGATTGGATTACTCGGCTGATCCCCCGGTTCAAACCGTAATTGCCAAGGACGTTCATGGTGAAATTTGGAAATTTAGGCATATTTACAGAGGAACGCCGAGGAGGCATTTGTTGACAACAGGGTGGAGTAGTTTTGTGAACCAGAAGAAACTTGTTGCTGGCGATTCAATTGTGTTCTTACGAGGGGCAGAGAATGGGGATTTGTGTGTTGGAATCAGACGGGCCAAGAGAGGAATTACTGGCGGCCCTGAGTCTCCCTCCGGATGGAATTCTGGCTCTGGAAATTGTGTTTCTTCATATGGGTCATTTTCAGTGTTTTTGAGGGAAGATGAGAACAAACTGATGAGAAATGGGTCTAATGGGAATTTGAACTCTGGTGGTGGGTTGAGGGGAAGGGGAAGAGTGAGGCCTGAAGTGGTTGTTGAAGCTGCAACACTTGCAGCTAATGGACAAACATTTGAAGTGGTTTATTACCCGCGGGCAAGCACTCCAGAGTTCTGCATTAAGGCTTCATCTGTGAGGGCTTCCATGAGGATTCAATGGTGCTCTGGGATGAGATTCAAAATGCCATTTGAAACCGAGGATTCTTCCCGGATTAGCTGGTTCATGGGAACCATAGCTTCTGTTCAGGTTGCTGACCCAATTCGCTGGCCTAATTCCCCATGGAGGCTTCTCCAGGTGACATGGGATGAACCAGATTTGCTGCATAATGTGAAGCACGTCAGCCCATGGCTGGTCGAGTTGGTATCAAACATGCCTGTCATTCACATGTCACCCTTTTCACCACCAAGAAAGAAATTGCGGTTTCCGCAACACCCCGACTTCCCTCTCGACGGCCAATTTTCAATGCCGTCATTTTCACGCAACCCCCTAGGGCCCAGCAGTCCTTTGTGTTGTCTATCCGACAACATTCCTGCAGGCATACAGGGAGCCAGGCATTCTCAATTTGGAATACCATTATCGGATCTCTACCTTCATAACAAACTGCAGTTGGATCTTTTCCCGTCCAGCTTGCAGCAGCTTGATCCGCATGGTAAATTATCCGATGGTTTGGTTTCAACCCACAAAAACAGCAATGACAATGTTTCTTGCTTGTTGACAATGGGGAATTCCAATAGTAAATCAGATAAAACTGATAATTTGAAGACACCCCAGTTTGTACTCTTTGGCCAACCAATACTGACTGAACAGCAGATCGCTTGTAGCTGTTCCAGTGATGCAGTCTCAAAAGTTGTAAATTTTCCAGAGAAGAAAGAAAGATCTGCATTTGACCAGCAATGGGTTCCAGAAAATCTGTCATGCGTTGCGTTTCCGTGGCGTCAAGGTTTTCACATGACTGAACTTGGCCTCGATACTGGTCATTGTAAGGTGTTTATGGAGTCGGAGTATGTAGGCCGGAATCTTGACCTCTCCGTTATTGGATCTTATGAAGAACTATACAAAAGGGTTGCAAACATGTTTGGAATAGAAAGATCAGAGATGCCATGTCATGTGCTATACCAGGATGCAACTGGTAATGTCAAACATACTGGAGATGAACCTTTCAGTGATTTCATGAAAACTGCAAAAAGATTGACAATTCTACTGGATTCAGGCAACAACTTTGGAAGGTAA

**ARF18-F: 5’-ATGGCTGGTCGAGTTGGTAT-3’**

**ARF18-R: 5’-GCATTGAAAATTGGCCGTCG-3’**

1. **ath-MIR472-p3_2ss6TG18AG TTTCCGACTCCGCCCATGCCAT**

**TRINITY_DN37745_c0_g2 F-box/FBD/LRR-repeat protein (FOL2)**

ATGAAGAGAAACACAAAGGAAATGCATGGATCAACTTCAGACATAATCAGTAATCTTCCAGAAAGTATAAAAGCAACAATCTTAGTGTGTTTGCCAATACAAGATGCGGTGAGGACTAGTGTTTTGTCAAGGAAATGGAGGTATATTTGGGTGAAACTTCCCCAACTTGTATTTGATTATACATTCTACAAAAAATCATTTCGGAAAACCAAGAATGAGCTTCTGATGATTATTTATCAAGTTCTGTTACTTCACCATGGACCGATACTCAAGTTCACCCTCTCTTTACCTAAACTGGAAAGCTGTTCTGAGATTGACCAGTTGATATATTTTGCATCAAAGAATGGTACCCAGGAATTCACCCTTCGCATTTGGACGGGTACTTATAAATTGCCATCATCACTGTTTTCATGTCTACAACTTAAGCATTTGAATCTCCATTCTTGCATGTTTAAGCCTCCGCCTGAATTTAAGGGATTTAGCAGACTTCTTAGCCTGGAGCTTCGCGAAGTCGTCATTACTGCTGACATATTGTCAAGTTTAATCTCCAGTTGCCCACTACTTGAACATCTGACAGTTGAAAGCTCCACCAGTTTTGAACACCTTGAAGTTGCTGCGCCTAATCTTAGATTCTTACGCTGTAAAGGTCTTTCC

**FOL2-F: 5’-TGCGGTGAGGACTAGTGTTT-3’**

**FOL2-R: 5’-TGAGTATCGGTCCATGGTGA-3’**

1. **gma-miR403a_R+1_2ss20TC21GT GCCGCTTAGATTCACGCACAAACTCTG**

**TRINITY_DN26124_c0_g4 protein argonaute 2 (AGO2)**

CAACTTATTTTCTGTGTGATGTCTGGAAAGCATGATGGTTACAAGTATCTCAAATGGGTGTCCGAGACCCAGATTGGTGTATTGACTCAATGTTGCTTAGTTGGTATTGCAAACAAAGGTAAAGACCAGGACCTTGCAAACATTGCTCTGAAGCTTAATTGCAAGCTTGGGGGTAGCAATGTAGAACTGAATGGACCACTCCCCGGTTTCGAAGGTGACGAACATGTTATGTTTGTGGGGGCTGATGTCAATCACCCTAAAGCATTTAATGCGTCTTGTCCGTCAATTGCAGCTGTTGTGGCATCAGTCAACTGGCCTGCTGCCAATCGTTATGTTGCTCGGATTTGCCCCCAAGAGCACCGGAAGGAGAAGATTGTGAATTTTGGGCCCATGTGTTTGGACCTAGTCAACACTTATGCTAAGCTTAACAAAGTCAAACCGAAGAAGATTGTTGTTTTTCGTGATGGGGTTAGTGAGGGCCAATTTGATATGGTTCTTAATGAAGAGTTACTGGATCTGAAGAATGCCATATATGAGGGAAACTACCGTCCTACGATCACTCTTGTTGTTGCCCAGAAGCGGCACCAGACTCGTTTGTTTCTTGAGAATGAGAGGGATGGGGGAGCATCTGGCAATGTGCCTCCGGGAACAGTCGTGGACACGACAATTGTTCATCCCTTTGAGTTTGATTTTTATCTGTGCAGCCATTATGGAAATCTCGGGACTAGCAAGCCAACCCACTACTATTGTTTGTTTGATGAGAACAAGTTCACTTCTGATAAGTTGCAGAAGCTCATATATGACTTGTGTTACACCTTTGCTCGATGCACCAAGCCTGTCTCGCTCGTCCCTCCTGTGTACTATGCTGATCTTGTTGCGTACCGAGGACGTCAGTATCAAGAGGTTGTCATGGAGTTTCAGCCACCTCCTTCAGTTTCATCTTCATCGTCATCATCTTCATCTTCTGTTGCTTCACTTAATGAGAGGTTCTATGCTTTGCACCCGGACCTCAAGGATACCATGTTTTTCATTTGA

**AGO2-F: 5’-GGCACCAGACTCGTTTGTTT -3’**

**AGO2-R: 5’- TAGTGGGTTGGCTTGCTAGT-3’**

1. **mtr-miR172a GCCGCGGAATCTTGATGATGCTGCAG**

**TRINITY_DN41667_c0_g2 Ethylene-responsive transcription factor RAP2-7 like (RAP2-7)**

GACAAGGCTGCAATAAAATGCAATGGAAGGGAAGCGGTGACCAACTTTGAGGCAAGCACATATGAAGAGGTGTTAAGCTCTGAGATTGAGAATGGAGGAAGCAGCCATAATCTAGACCTCAACTTGGGTATTTCTACCCCTTGTTTTGCTAATGGCCAAATGGGAACCAATGACTTGAGCAGTGGTTTACATTTGCAGAGTGACTTGAGCAATATGCATGAGAACAGAATGGCTGAGAACTCCGTTACAACAATGGGAACTCAACTACCTCATGGCCAAATAATGGTTTCTGAGCACCCTCCTCTCTGGAAAGGTGTAAATTCCCATGCCGTTCCCATCTATGAGGGAAGAGCAATAGAGAATAGTATGGAATTTGATTCTTCACCGAGCTGGGGATTGCAATTCCAAGGCCCATTTGGTGGTGACGTTCCACTGCCACTCTTCTCTACTGCAGCATCATCAGGATTCGCTACTTCAACCATCGCTACTTCATCAGCTGCGGTTTACCAACCTTGGTTTCCAACTACAACAATCCCCCACAACCATTACTTTCCATCGATCATCGATTCTAACAACATATCTCAGTACTACTGCAGGAGCTGA

**RAP2-7-F: 5’-AGAATGGCTGAGAACTCCGT-3’**

**RAP2-7-R:5’-TTGGAATTGCAATCCCCAGC-3’**

1. **ptc-miR160h_1ss15CG TGCCTGGCTCCCTGGATGCCA**

**TRINITY_DN39928_c3_g4 Auxin response factor like (ARF22)**

ACCCAATCGGATGCTAACAATGGTGGAGGGTTTTCGGTTCCACGTTATTGTGCAGAGACGATCTTTCCACGGTTGGATTACACTGCTGATCCTCCTGTTCAGACAATCCTTGCAAAGGATGTTCACGGTGAGACTTGGAAATTTCGTCATATTTATAGGGGAACACCAAGGCGCCATCTTCTGACAACAGGATGGAGCACTTTTGTGAACCACAAGAAGCTTGTTGCAGGGGATTCGATTGTGTTCTTGAGAGCAGAAAATGGGGATCTCTGTGTTGGAATCCGGCGAGCAAAGAGGGGAATTGGAGGTGGGCCCGAGTCGTCATCCGGATGGAATCCTGCCGGTGGTAATTGTGTCATACCATATGGAGGGTTTTCCGCCTTTTTGAGGGACGATGAGAACAAATTAATGAGAAATGGTAATGGGGGAAATTCGAGTGGTAGTGGGAGCTCAACCGGAAAGGGGAAAGTCAGGCCTGAATCGGTTGTCGAAGCTGCAACACTGGCAGCCAACAAACAGCCCTTTGAGGTTGTTTACTATCCACGAGCGAGTACTCCGGAGTTCTGTGTGAAAGCCTCACTTGTGAAGGCTGCGTTGCAGATCTGCTGGTGCTCGGGAATGAGGTTCAAGATGGCCTTCGAAACCGAGGATTCTTCCCGGATAAGTTGGTTCATGGGAACCATATCTTCTGTTCAGGTTGCCGATCCCATTCGCTGGCCCGATTCGCCTTGGAGACTTCTCCAGGTGACATGGGATGAGCCTGATTTGCTTCAAAACGTCAAACACGTAAGCCCATGGCTAGTGGAATTGGTCTCAAACATGCCCGTGATCCATCTCTCCCCATTTTCACCTCCGCGTAAGAAGTTGAGACTACCCCTACACCCCGATTTCCCTCTCGACGGCCAACTTGCGATGCCAACATTTTCCGGCAACCCGTTACGTCCCTTCAGTTGTCTTCCCGACAATGCTCCTGCTGGCATGCAGGGAGCCAGGCATGCTCAATACGGTTTATCTTTATCCAATCTACACCTCAATAAACTACACTCGGGCCTGTTTTCAGCCAATTTCCCAACATTAGATAGTGTTGCTGCAATGAAAATTAGACCCTCCAATCCTGCTATCACCCAAAAGCCTAGCGGTGGCGAAAATGTTTCTTGCTCACTAACTATTGGGAATTGTACTCCTAATTCAAAGAAACCGGACAATGGGAAAGGAACCCAATTCGTGCTTTTCGGTCAACCAATACTTACTGAGAAACAGATCTCTCTAAGCTGCTCCGGAAATAGTTCCTCAGATGGGAATGCAGATAAGATTGCAAATATTTCTGATGGTTCTGGTTCTGCACTTCATCAACCGGAGCAGAGATCTTCTTGCAAAGGGATTCTATGGGATAAAGATAATCATCGAGACAAAGAACTCAGCTTGGAAATTGGTCATTGTAAGGTATTTATGGAATCAGAGGATGTGGGTCGTACTCTTGATCTTTCAATGCTTGGGTCTTATGAAGAATTGTAC

**ARF22-F:5’-TGCTAACAATGGTGGAGGGT-3’**

**ARF22-R:5’- CACCGTGAACATCCTTTGCA-3’**

1. **ath-miR858b GCCGTTCGTTGTCTGTTCGACCTTG**

**TRINITY_DN31256_c0_g4 Myb-related protein (MYB6)**

CCCTTCTCTCTCTCTGCATTTCCATTGGGTTTTTGGAATAGTACAACAATGAGGAAGCCTTGCTGTGACAAACAGGACACCAACAAGGGCGCTTGGACCAAGCAAGAAGACCAAAAGCTTATTGATTACATTCAAACTCATGGCGAAGGTTGTTGGCGCACCCTTCCCAAGGCTGCCGGGTTGCATCGTTGTGGTAAAAGTTGCAGACTGAGGTGGATAAACTATCTTAGGCCAGACATCAAACACGGCAACTTTGGTCAAGATGAAGAAGACCTCATCATCAAACTCCATGCCCTCCTTGGCAACCGGTGGTCATTGATATCCGGAAGATTACCAGGGCGAACAGACAATGAAGTGAAGAACTACTGGAACACTCACCTGAAGCGAAAGCTGATACGTGTGGGTATTGATCCAGATAACCATCGATTGAACCAAACACTTCCTCGCCTACAAAACCAG

**MYB6-F:5’-CCAGACATCAAACACGGCAA-3’**

**MYB6-R:5’-TGTCTGTTCGCCCTGGTAAT-3’**

1. **mtr-MIR2592ao-p5_2ss1AT18TC GCCGCGTTTCCAACTGCAACAATC**

**TRINITY_DN26936_c0_g1 WRKY transcription factor (WRKY53)**

ATGGAGAATGGTTTGAATTGGGAACAAACACTGATCAATGAGCTGGCTCAAGGGATGGAGCTAGCCAAACAACTAAGAGCTCACTTGAATTCAACTTCCTCTGCCGAAAGTCGAGAAATTTTACTACAGAGGATACTATCTTCATACGAGAAGGCCTTTATGATTCTCAAGTGGAGTAGATCAATTGGACATCCTCAGTTATCGGCACCCATCACCAATTTGCCAGAGTCTTCAATTTCTGGCGACGGGAGTCCTCAGAGCGAGGAATTCGACCGGGGTTTTAAAGATCAGCAGGACCAGAATGATGTTTCCAAGAAGAGAAAGGTGTTGCCCACATGGACAGGACAAGTGAGGGTTTGCTCTGAGAATGGGTTTGAAGGTCCCACGGAGGATGGTTACAGTTGGAGAAAATATGGACAGAAAGACATTCTTGGCGCCAAATATCCCAGAAGCTACTACAGATGCACATACCGCAGCCTTCAAAACTGCTGGGCTACAAAGCAAGTCCAACGATCGGACGACGATCCCACCATCTTCGAAATTATCTACAGAGGGAGACACAGTTGCAACCAAACCCCACACACAGCTCCACCACCACCACCATCA

**WRKY53-F:5’-CACCCATCACCAATTTGCCA-3’**

**WRKY53-R:5’-AGCAAACCCTCACTTGTCCT-3’**

1. **mtr-miR171d GTGATTGAGCCGTGCCAATATC**

**TRINITY_DN38636_c0_g1 Scarecrow-like protein (SCL6)**

ATGATAGGAATGCAATTTAATTTGCAAGATAAGGGTTTGTTGGAAGTTACAGGTTTCGATTCTTCGGAAACAAAGTGGAAGAAGGGAATCAGTTGCGCGAGCAATGAACCCATCTCTGTTCTTGACACCAGGAGGAGTCCCAGCCCTTCTACTTCCACATCCACCCTCTCTTCCTCTCTCGGCGGCGGCGCAACCGCCGTCTGCGGCGGTTCCGGCGACAACACAGCAAGTCTGGCGGCGGTTTCCGACACAATCCACCACCAGAAATGGCCGGAGAGTGGCCCGGTTGGCTTGACAGAGCCCGCCGGCGAATGGGCGTCGGAGCTGCAGCCGATTCCGGCGGCCCTGGAGGTTTCCGGCGGCGAAAGATTTGGGATTGGGTTGGAAGATTGGGAGAGTTTGTTGTCTGAATCGGGTCAAGATCAGTCTATTCTCCGGTGGATTGCAACCGAATTCGACGGCGGCAATGTCGCCCTCGCTGCCGTCGTCGATCAGGGTTCCACCGCCGTTGACGGTGGCTCTAATGGTGGTTCTGGTTCATCTTCATCTACGCCAAAAATTTCTGCTGAAACAGGTAATCCCAGAGCTTCAAATCTTGGGTTCAACAACAACTCACAAATCCCCATTTTGGGTTCTTCAATCAACAATCTATTTTCTAGTGATTTGATCTACCAGCTTGAAAACCCAGAGTCAAAGCCTCAGATTTTCAATCCACAAGTATTCATAAACCAACACCAACTTTCTCATCTGTCTCAGACTCAAATTCAAACACAACCCTCTCAACACCATCTTCAACAAACACCATTAATGGCTATATACGACCAGCTTTACAAAGCGGCAGAGCTGATACAATCTGGGAACTTCTCTCTCGCGCAAGAGATATTGGCGCGGCTCAATCACCAGCTCTCTTCCTCTTTGTCAAAACCCTTTCAAAGGTCTGCTTTTTACTTCATGGAAGCTCTTCAGCTCTCTCTTCTTATGCTCAACCATGTCAATCCTTTGCCTCCTAGAACTCCTACGCCCTTTGATGCTATGTTCAAAATGGGTGCTTATAAATTGCTCTCTGAAGCCTCACCTGTTACCCATTTCATGAATTTCACTTCTAACCAAGCAATCCTCGAAGCTCTTGGCTATGGCGGACGTGTTCATATCATCGATTTCGATATTGGGTTTGGTGCTCAATGGGCTTCTTTGATGCAAGAGCTTCCAAGCAGGAACAGAGGCAAAACCCCATTTTTGAAAATCACTGCCTTCGCCTCTCCTTTAACCCACCATCCTCTCGAG

**SCL6-F:5’-AACCCAGAGTCAAAGCCTCA-3’**

**SCL6-R:5’-TGGTGTTGAGAGGGTTGTGT-3’**
