# Supplementary material for: Integration of small RNA, degradome, and transcriptome sequencing data illustrates the mechanism of low phosphorus adaptation in Camellia oleifera
Source: Front Plant Sci. 2022 Aug 1;13:932926. doi: 10.3389/fpls.2022.932926 (PMC9377520; doi:10.3389/fpls.2022.932926)
Supplement: Supplementary file 1 [file Data_Sheet_1.ZIP › Supplementary materials/Supplementary Table S3 Overview of sRNA sequencing.docx]

**Table S2 Overview of sRNA sequencing**

| **Category** | **0d1** | **0d2** | **0d3** | **1d1** |
| --- | --- | --- | --- | --- |
| **Raw reads** | 19,018,590 | 20,064,010 | 24,814,201 | 22,004,386 |
| **Adapter and length filter** | 2,877,191 | 1,616,441 | 4,437,645 | 8,124,616 |
| **Junk reads** | 112,334 | 101,458 | 285,145 | 64,059 |
| **Rfam** | 1,459,027 | 1,673,554 | 4,083,187 | 2,328,987 |
| **Repeats** | 13,850 | 12,013 | 17,059 | 30,915 |
| **Valid reads** | 14,562,710 | 16,666,510 | 15,999,525 | 11,465,044 |
| **Category** | **1d2** | **1d3** | **3d1** | **3d2** |
| **Raw reads** | 15,917,026 | 21,966,203 | 17,113,534 | 16,584,491 |
| **Adapter and length filter** | 4,669,220 | 5,038,518 | 8,327,877 | 2,910,542 |
| **Junk reads** | 49,026 | 77,408 | 36,661 | 74,438 |
| **Rfam** | 1,773,865 | 2,555,938 | 1,537,842 | 1,317,755 |
| **Repeats** | 20,828 | 24,633 | 20,195 | 12,452 |
| **Valid reads** | 9,412,453 | 14,280,458 | 7,198,931 | 12,274,110 |
| **Category** | **3d3** | **7d1** | **7d2** | **7d3** |
| **Raw reads** | 10,272,974 | 26,154,739 | 14,267,524 | 13,348,028 |
| **Adapter and length filter** | 5,846,735 | 4,417,524 | 7,139,288 | 6,637,604 |
| **Junk reads** | 21,521 | 115,208 | 37,276 | 22,156 |
| **Rfam** | 845,974 | 1,866,062 | 1,221,740 | 1,514,201 |
| **Repeats** | 9,640 | 21,302 | 16,700 | 20,605 |
| **Valid reads** | 3,553,062 | 19,743,484 | 5,860,793 | 5,164,051 |
| **Category** | **30d1** | **30d2** | **30d3** |  |
| **Raw reads** | 23,047,909 | 20,502,806 | 11,637,581 |  |
| **Adapter and length filter** | 6,360,526 | 3,179,390 | 1,753,467 |  |
| **Junk reads** | 77,701 | 162,823 | 95,947 |  |
| **Rfam** | 2,337,222 | 2,187,414 | 1,003,154 |  |
| **Repeats** | 24,207 | 14,929 | 10,820 |  |
| **Valid reads** | 14,258,496 | 14,964,466 | 16,554,826 |  |
