# Supplementary material for: Integration of small RNA, degradome, and transcriptome sequencing data illustrates the mechanism of low phosphorus adaptation in Camellia oleifera
Source: Front Plant Sci. 2022 Aug 1;13:932926. doi: 10.3389/fpls.2022.932926 (PMC9377520; doi:10.3389/fpls.2022.932926)
Supplement: Supplementary file 1 [file Data_Sheet_1.ZIP › Supplementary materials/Supplementary Table S7 The profiles of novel miRNAs.docx]

**Table S6 Profiles of novel miRNAs**

| Novel miRNAs | Mature sequence (5' to 3') | Length | dG | MFEI |
| --- | --- | --- | --- | --- |
| PC-5p-559054_17 | ACTATCACTGAATATCACCTTACC | 24 | -93.4 | 1.5 |
| PC-3p-1030661_6 | AAGGTGATATTCAGTGATGGTTGA | 24 | -93.4 | 1.5 |
| PC-5p-559054_17 | ACTATCACTGAATATCACCTTACC | 24 | -92.2 | 1.6 |
| PC-5p-320396_39 | TGAGGGATTTGAGAAGGGATT | 21 | -60 | 1.2 |
| PC-3p-30509_767 | AATCCCTTCTCAAATCCTCCAACC | 24 | -60 | 1.2 |
| PC-5p-334370_37 | AATGATCATGAAAGATTTGTGACC | 24 | -69.8 | 1.5 |
| PC-3p-24651_983 | TCATAAATCTTTCATGATCAT | 21 | -69.8 | 1.5 |
| PC-5p-310518_41 | TTTGGTTGGGAATTTGGGAAG | 21 | -45 | 1 |
| PC-3p-634590_13 | TTCCCAAATCCCTCAACCAAACAC | 24 | -45 | 1 |
| PC-5p-674588_12 | CTGTGTTTGGTTGGGGGATTT | 21 | -67.2 | 1 |
| PC-3p-90001_209 | AAATCTCCCAACCAAACACAGCAT | 24 | -67.2 | 1 |
| PC-5p-173050_91 | ACAGAATGAGACACAGATGTGAGA | 24 | -62 | 1.4 |
| PC-3p-1123841_6 | CACATCTGTGTCCCATTTTGT | 21 | -62 | 1.4 |
| PC-5p-24863_974 | GGGATTGGTGGCTTGAAAAGC | 21 | -73 | 1 |
| PC-3p-2177_9260 | NTTCCAAGTCCACCCATTCCTA | 22 | -73 | 1 |
| PC-5p-134341_127 | TATTGTGTGGTTGAGATTTAC | 21 | -63.9 | 1.7 |
| PC-3p-33289_692 | ATCTCAACCACACAATGCACCACT | 24 | -63.9 | 1.7 |
| PC-5p-28038_848 | ATTGTGTGGTTGAGATTTAATGAC | 24 | -66.7 | 1.7 |
| PC-3p-238290_59 | ATTAAATCTCAACCACACAATGCA | 24 | -66.7 | 1.7 |
| PC-5p-1219212_5 | ATACTGTGTTTGGTTGGGGGA | 21 | -45.6 | 1.5 |
| PC-3p-242493_58 | AAATCCCCCAACCAAACACAGTGT | 24 | -45.6 | 1.5 |
| PC-5p-156691_104 | ATTTTGTGGTTCAAATGGCTC | 21 | -84.6 | 1.6 |
| PC-3p-115339_154 | ATTTGAGCCATTTGAGTCACAAAA | 24 | -84.6 | 1.6 |
| PC-5p-159142_102 | TCAACCACACAATACACCACT | 21 | -53.8 | 1.1 |
| PC-3p-130040_132 | AGTGGTGCATTGTGTGGTTGAGAT | 24 | -53.8 | 1.1 |
| PC-5p-322224_39 | CAGCGGTGTGTTAAGAATATG | 21 | -38.6 | 1.6 |
| PC-3p-845853_8 | TTATTTTCTTGACACATCACT | 21 | -38.6 | 1.6 |
| PC-5p-33289_692 | ATCTCAACCACACAATGCACCACT | 24 | -45.5 | 1 |
| PC-3p-193232_79 | AAGTGGTGTATTGTGTGGTTG | 21 | -45.5 | 1 |
| PC-5p-115327_154 | AAACAAATGACCAAATGTCTCACA | 24 | -57.5 | 1.3 |
| PC-3p-528113_18 | ACATTTGGTCATTTGTTTGGTAGA | 24 | -57.5 | 1.3 |
| PC-5p-1402690_5 | TCAAATGGTCAAAACGATGTCGTT | 24 | -69.2 | 1.3 |
| PC-3p-18140_1384 | AACGACGTCGTTTTGGCCATTTGA | 24 | -69.2 | 1.3 |
| PC-5p-317517_40 | GGGATTTGGAAAGAGAAA | 18 | -47.9 | 0.9 |
| PC-3p-132795_129 | CCTTTCCCTTTCCAAATTCCT | 21 | -47.9 | 0.9 |
| PC-3p-1055017_6 | TGATAGGGGTTACTTGGGGA | 20 | -47.4 | 1.4 |
| PC-3p-346339_35 | TAATGTTTTCAAAACTCGA | 19 | -76.8 | 1.3 |
| PC-5p-542248_18 | TTTGGATAGGAATTTGGAAAG | 21 | -57.9 | 0.9 |
| PC-3p-656142_13 | TCCGCCTGCACTTCCATGA | 19 | -50.1 | 1 |
| PC-5p-865400_8 | GGGACCAAGAACTCAGACACA | 21 | -61.3 | 1.3 |
| PC-3p-408527_27 | CCTTGCACTTTTCTCTGCACA | 21 | -66.8 | 1.2 |
| PC-5p-148677_112 | TATGGGTGTCCGACATGTGTC | 21 | -41.3 | 1 |
| PC-3p-549768_17 | CTTCTCCACGTTCTTTCTCCG | 21 | -69.3 | 0.9 |
| PC-3p-18516_1353 | AGAGATTATTTTCGAGCCATTCGT | 24 | -52.9 | 1.3 |
| PC-3p-47_152707 | GAATGTTGGGTCCTTTGAGGTCA | 23 | -46.3 | 1.2 |
| PC-3p-66331_303 | TCTCTTTCCCTTTCCAAATCC | 21 | -52.1 | 0.9 |
| PC-3p-545895_17 | TGTGGTGAGAGTGAGAGA | 18 | -25.5 | 1 |
| PC-3p-99538_185 | TTCCCTCCACAAATCCAAGAT | 21 | -51.7 | 1.2 |
| PC-5p-92854_201 | CTTGCACGGAGATAGAATACAAAA | 24 | -48.5 | 0.9 |
| PC-3p-100894_182 | TGATTTTAGATTGAGTGTTT | 20 | -17.1 | 0.9 |
| PC-3p-72191_274 | TTCACACACCTTTTTCCTTTCCCT | 24 | -34.3 | 1.2 |
| PC-3p-157094_104 | ATAACCCACTTCCCAAAATAGGCC | 24 | -48.1 | 1 |
| PC-3p-523015_19 | AACGGTGCAGATGGTTTTCATGAT | 24 | -45.3 | 1.4 |
| PC-3p-626112_14 | TTTCCTTTCCTTTTCTAAATCCCT | 24 | -75 | 1.3 |
| PC-5p-174389_91 | AGTCAACCCGCAACCGCCCGCAGT | 24 | -52.8 | 1 |
| PC-3p-418202_26 | GTTGTAACTTGCCTTGATATTTGG | 24 | -44 | 1 |
| PC-3p-41728_531 | TCAAATCCCTTCTCAAATCCC | 21 | -51.2 | 1.1 |
| PC-3p-958919_6 | TGATCCAACAGTTGTAAGTGT | 21 | -60 | 1.2 |
| Average |  | 22.21 | -56.36 | 1.22 |
