# Supplementary material for: Integration of small RNA, degradome, and transcriptome sequencing data illustrates the mechanism of low phosphorus adaptation in Camellia oleifera
Source: Front Plant Sci. 2022 Aug 1;13:932926. doi: 10.3389/fpls.2022.932926 (PMC9377520; doi:10.3389/fpls.2022.932926)
Supplement: Supplementary file 1 [file Data_Sheet_1.ZIP › Supplementary materials/Supplementary Table S9 Predicted Camellia oleifera mRNA targets for the novel miRNAs.docx]

| miRNA ID | Locus target | Target description | miRNA/mRNA pairing |
| --- | --- | --- | --- |
| PC-3p-100894_182 | TRINITY_DN30301_c0_g3 | LRR receptor-like serine/threonine-protein kinase | - - - \| \| \| \| - \| \| \| \| \| \| \| \| \| \| \| \| \| |
| PC-3p-132795_129 | TRINITY_DN33563_c0_g5 | unnamed protein product, partial | - - - : : - - \| \| \| \| \| \| \| \| \| \| \| \| \| \| |
| PC-3p-132795_129 | TRINITY_DN35456_c0_g2 | Calcineurin-binding protein like | : \| \| : \| \| \| \| \| \| \| : \| \| \| \| - \| \| \| \| |
| PC-3p-18140_1384 | TRINITY_DN31490_c2_g1 | NA | \| \| : \| \| \| \| - : : \| \| \| \| \| \| \| \| \| \| \| \| \| \| |
| PC-3p-27079_883 | TRINITY_DN42730_c0_g2 | NA | \| \| \| \| \| \| \| \| \| \| \| \| \| \| - : \| \| \| \| \| \| \| \| |
| PC-3p-30509_767 | TRINITY_DN42730_c0_g2 | NA | \| \| \| \| \| - : \| \| \| \| \| \| \| \| \| : \| \| \| \| \| \| \| |
| PC-3p-346339_35 | TRINITY_DN33230_c0_g2 | Branched-chain-amino-acid aminotransferase | - \| - : \| \| \| \| \| \| \| \| \| : \| \| \| \| \| |
| PC-3p-346339_35 | TRINITY_DN42258_c0_g3 | Ankyrin repeat-containing protein | \| \| \| \| - \| \| - \| \| \| \| \| \| : \| : \| \| \| |
| PC-3p-41728_531 | TRINITY_DN28460_c0_g1 | U3 small nucleolar ribonucleoprotein protein MPP10 | - \| \| \| \| \| \| \| - \| \| \| \| \| \| - \| \| \| \| \| |
| PC-3p-41728_531 | TRINITY_DN34544_c0_g6 | Glycerol-3-phosphate transporter 1 like | - \| \| \| \| \| \| : - \| \| \| \| \| \| \| \| \| \| \| |
| PC-3p-41728_531 | TRINITY_DN38304_c0_g1 | zeaxanthin epoxidase | \| \| - : \| \| \| \| \| \| \| \| \| \| - \| \| \| \| \| \| |
| PC-3p-44318_495 | TRINITY_DN40647_c2_g5 | NA | - \| \| \| \| \| \| - \| \| \| \| \| \| \| \| \| \| \| : \| |
| PC-3p-44318_495 | TRINITY_DN42805_c2_g2 | Methyl-CpG-binding domain-containing protein | \| - \| \| \| \| - - \| \| \| \| \| \| \| \| \| \| \| \| \| |
| PC-3p-459616_23 | TRINITY_DN38590_c0_g4 | hypothetical protein MANES_08G157000 | - - - - : \| \| \| \| \| \| \| \| : \| \| \| \| \| |
| PC-3p-545895_17 | TRINITY_DN34459_c0_g1 | proteasome subunit alpha type-2-A | \| \| - : \| \| - \| \| \| \| \| \| \| \| \| \| \| |
| PC-3p-545895_17 | TRINITY_DN45451_c1_g1 | NA | \| \| \| \| \| \| \| \| - \| \| \| \| \| \| \| : \| |
| PC-3p-545895_17 | TRINITY_DN46149_c1_g4 | NA | \| \| \| \| \| \| \| \| -\| \| \| \| \| - \| \| \| \| |
| PC-3p-66331_303 | TRINITY_DN30968_c2_g4 | NA | \| \| \| - - \| - \| \| \| \| \| \| \| \| \| \| \| \| \| \| |
| PC-3p-66331_303 | TRINITY_DN44551_c1_g4 | AMP deaminese | - - - - - \| \| \| \| \| : \| \| \| \| \| \| \| \| \| \| \| |
| PC-3p-90001_209 | TRINITY_DN42730_c0_g2 | NA | \| \| \| \| \| \| \| \| \| \| \| \| \| \| \| \| |
| PC-5p-182503_85 | TRINITY_DN34544_c0_g6 | Glycerol-3-phosphate transporter 1 like | \| \| \| \| - \| \| \| \| \| \| \| \| \| \| - \| : \| \| \| \| \| \| |

Table S8 Predicted *Camellia oleifera* mRNA targets for the novel miRNAs

“|” indicates a Watson-Crick base pairing; “:” is a G:U base pairing, and “-” indicates a mismatch.
