# Supplementary figures and images for: Integration of small RNA, degradome, and transcriptome sequencing data illustrates the mechanism of low phosphorus adaptation in Camellia oleifera
Source: Front Plant Sci. 2022 Aug 1;13:932926. doi: 10.3389/fpls.2022.932926 (PMC9377520; doi:10.3389/fpls.2022.932926)

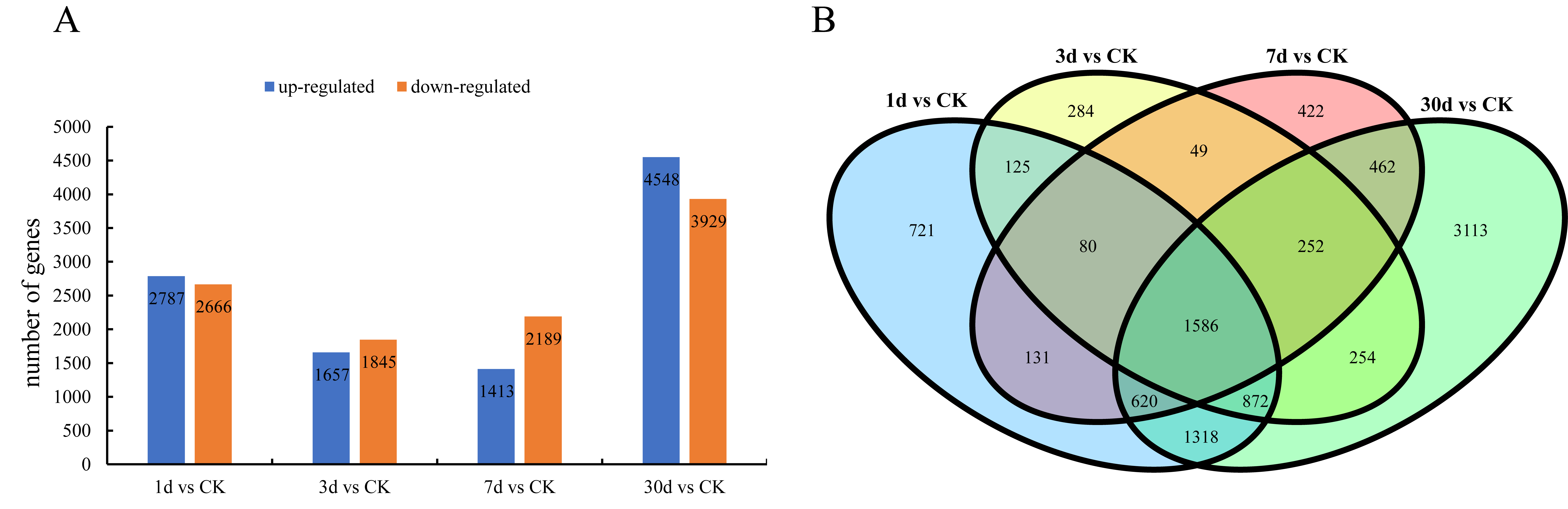

Supplement: Supplementary file 1 [file Data_Sheet_1.ZIP › Supplementary materials/Fig. S1 Expression profiling of –Pi-responsive DE mRNAs in C. oleifera.tif]

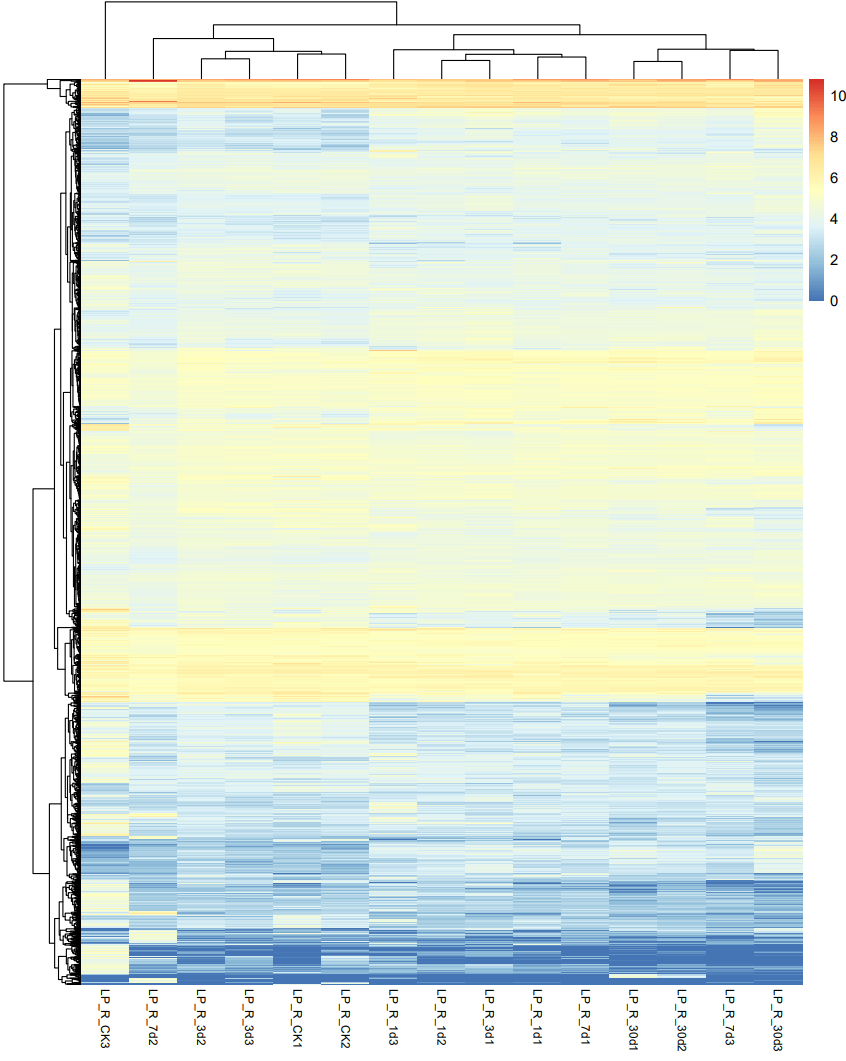

Supplement: Supplementary file 1 [file Data_Sheet_1.ZIP › Supplementary materials/Fig. S2 The heatmap of gene expression.tif]

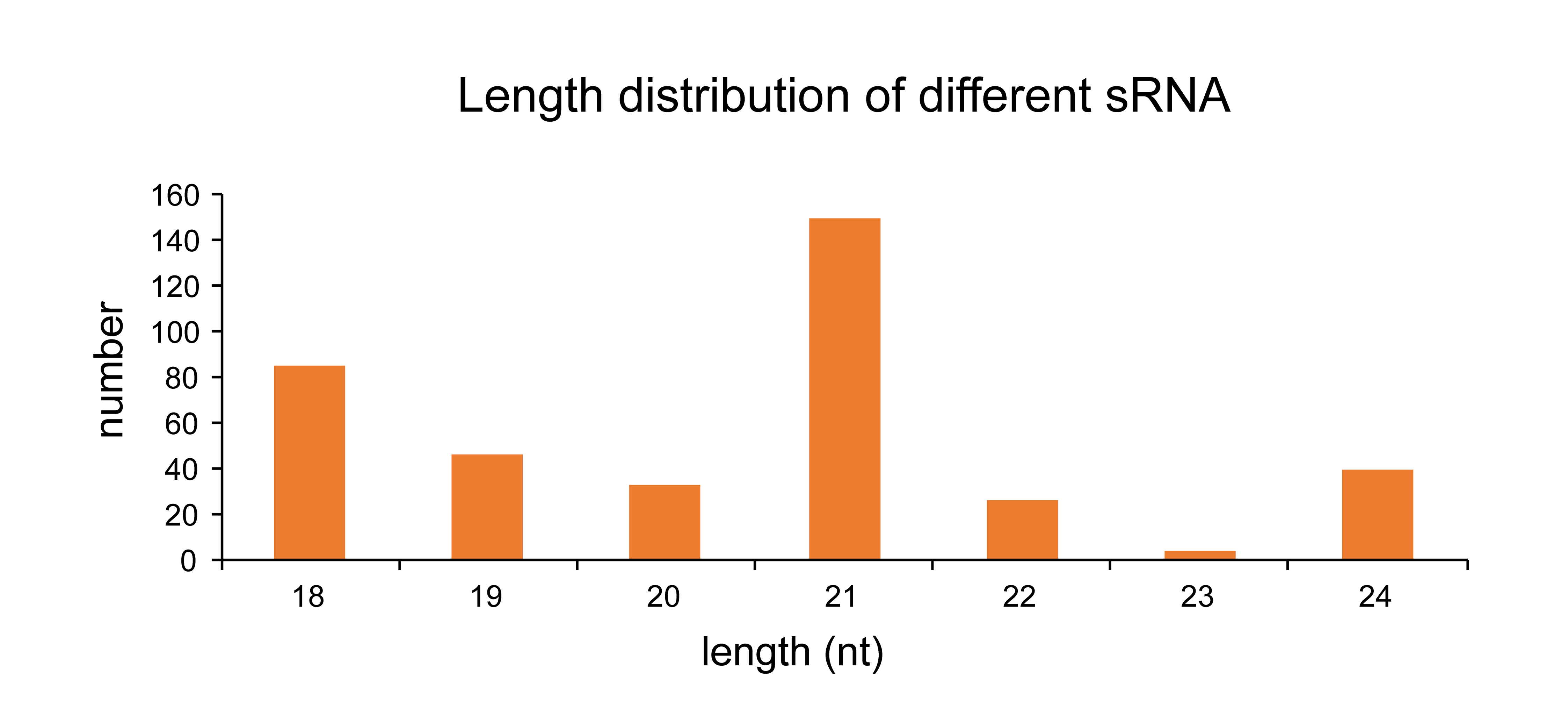

Supplement: Supplementary file 1 [file Data_Sheet_1.ZIP › Supplementary materials/Fig. S3 Length distribution of different sRNA.tif]

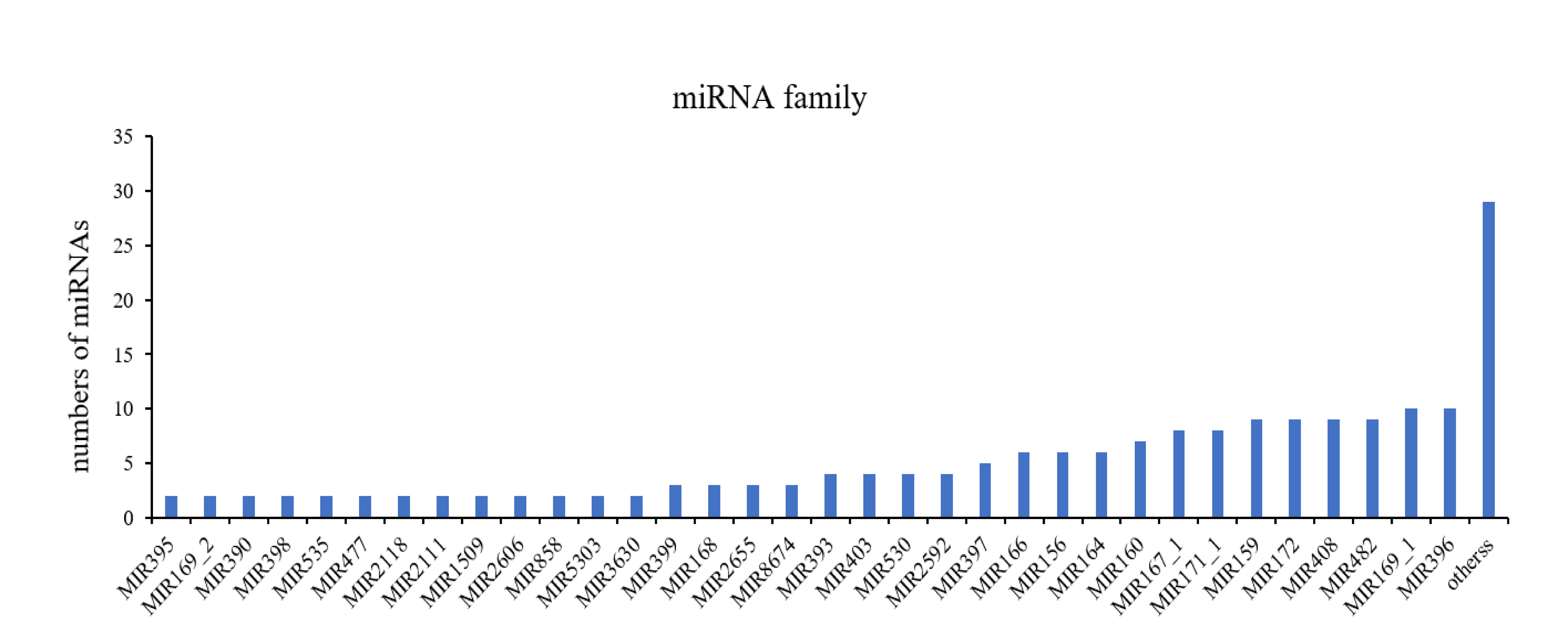

Supplement: Supplementary file 1 [file Data_Sheet_1.ZIP › Supplementary materials/Fig. S4 Different miRNA families .tif]

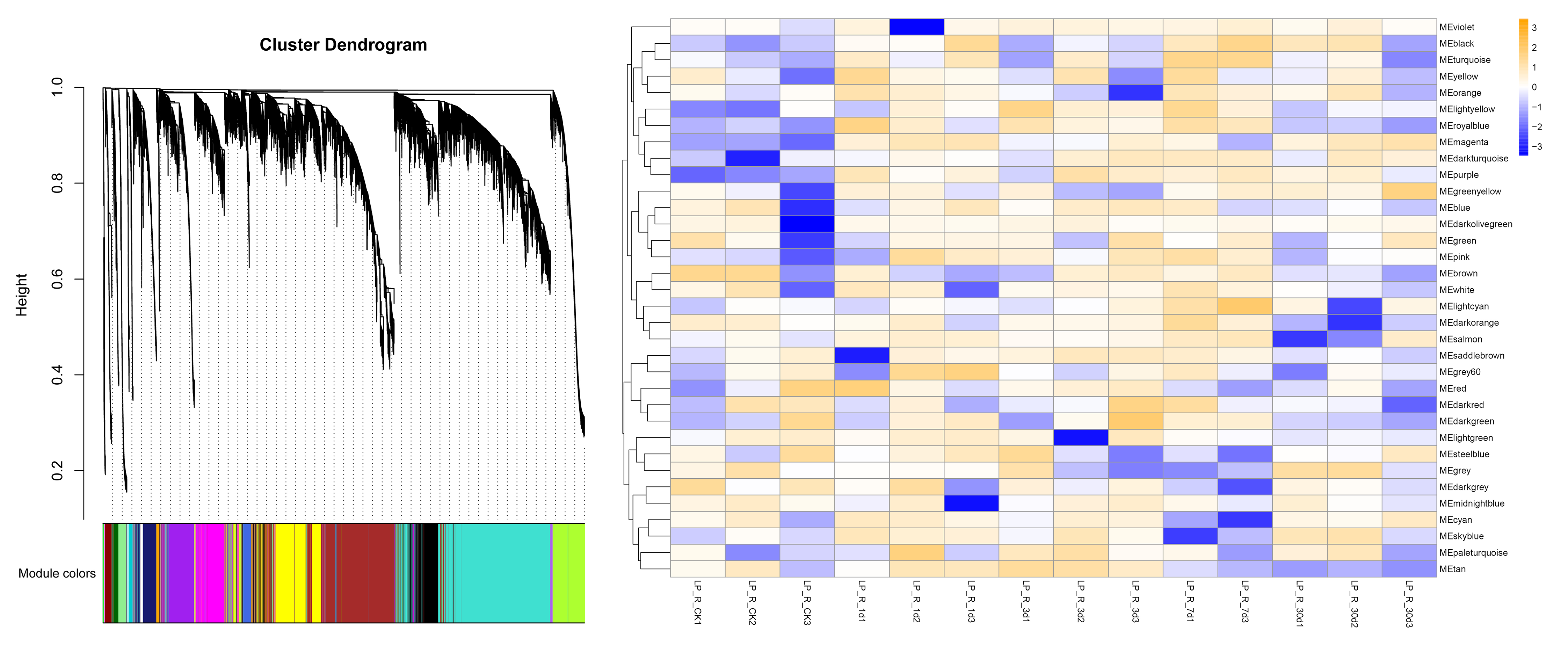

Supplement: Supplementary file 1 [file Data_Sheet_1.ZIP › Supplementary materials/Fig. S5 The 34 gene expression modules.tif]
