# Supplementary material for: Integration of small RNA, degradome, and transcriptome sequencing data illustrates the mechanism of low phosphorus adaptation in Camellia oleifera
Source: Front Plant Sci. 2022 Aug 1;13:932926. doi: 10.3389/fpls.2022.932926 (PMC9377520; doi:10.3389/fpls.2022.932926)
Supplement: Supplementary file 1 [file Data_Sheet_1.ZIP › Supplementary materials/Supplementary Table S6 Length distribution of unique miRNA.doc]

| length | Known miRNA | | Novel miRNA | |
| --- | --- | --- | --- | --- |
|  | Number | % | Number | % |
| 18 | 83 | 26.10 | 2 | 2.94 |
| 19 | 44 | 13.84 | 3 | 4.41 |
| 20 | 31 | 9.75 | 2 | 2.94 |
| 21 | 124 | 38.99 | 26 | 38.24 |
| 22 | 25 | 7.86 | 2 | 2.94 |
| 23 | 2 | 0.63 | 2 | 2.94 |
| 24 | 9 | 2.83 | 31 | 45.59 |
| All | 318 | 100.00 | 68 | 100.00 |

**Table S5 Length distribution of unique miRNA**
